# The future of critical care: AI-powered mortality prediction for acute variceal gastrointestinal bleeding and acute non-variceal gastrointestinal bleeding patients

**DOI:** 10.3389/fmed.2025.1580094

**Published:** 2025-05-16

**Authors:** Zhou Liu, Guijun Jiang, Liang Zhang, Palpasa Shrestha, Yugang Hu, Yi Zhu, Guang Li, Yuanguo Xiong, Liying Zhan

**Affiliations:** ^1^Department of Intensive Care Unit, Renmin Hospital of Wuhan University, Wuhan, China; ^2^Department of Radiology, Renmin Hospital of Wuhan University, Wuhan, China; ^3^Department of Ultrasound, Renmin Hospital of Wuhan University, Wuhan, China; ^4^Department of Pharmacy, Renmin Hospital of Wuhan University, Wuhan, China

**Keywords:** acute variceal gastrointestinal bleeding, acute non-variceal gastrointestinal bleeding, extremely randomized trees, gradient boosting, artificial intelligence, mortality

## Abstract

**Background:**

Acute upper gastrointestinal bleeding (AUGIB) is one of the most common critical diseases encountered in the intensive care unit (ICU), with a mortality rate ranging from 15 to 20%. Accurate stratification of acute gastrointestinal bleeding into acute variceal gastrointestinal bleeding (AVGIB) and acute non-variceal gastrointestinal bleeding (ANGIB) subtypes is clinically essential as distinct entities require markedly different therapeutic approaches and even divergent prognostic implications. AUGIB characterized by hemorrhagic shock, hypotension, multiple organ dysfunction (MODS), and even circulatory failure is life-threatening. Machine learning (ML) prediction model can be an effective tool for mortality prediction, enabling the timely identification of high-risk patients and improving outcomes.

**Methods:**

A total of 3,050 acute upper gastrointestinal bleeding (AUGIB) patients were included in our research from the MIMIC-IV database, among which 625 patients were classified as AVGIB and 2,425 patients were categorized as ANGIB. Patients’ clinical features, intervention methods, vital signs, scores, and important laboratory results were collected. The Synthetic Minority Over-sampling Technique-Edited Nearest Neighbors (SMOTE-ENN) and Adaptive Synthetic Sampling (ADASYN) were adopted to address the imbalance of the dataset. As many as 12 machine learning (ML) algorithms, namely, logistic regression (LR), decision tree (DT), random forest (RF), gradient boosting (GB), AdaBoost, XGBoost, Naive Bayes (NB), support vector machine (SVM), light gradient-boosting machine (LightGBM), K-nearest neighbors (KNN), extremely randomized trees (ET), and voting classifier (VC), were performed. The model performance was evaluated using accuracy, precision, recall, F1-score, and area under the receiver operating characteristic curve (AUC). Shapley Additive exPlanations (SHAP) analysis was conducted to identify the most influential features contributing to mortality prediction.

**Results:**

In terms of AVGIB patients, extremely randomized trees model demonstrated excellent predictive value among other ML models, with the AUC of 0.996 ± 0.007, accuracy of 0.996 ± 0.009, precision of 0.957 ± 0.024, recall of 0.988 ± 0.012, and F1 score of 0.972 ± 0.007. The top 10 primary feature variables of ET model were whether combined with acute kidney failure, transfusion of albumin, vasoactive drugs, transfusion of plasma, transfusion of platelet, the max of international normalized ratio (INR), the max of prothrombin time (PT), and the max of activated partial thromboplastin time (APTT). In case of ANGIB patients, gradient boosting model proven to be the optimal machine learning models, with the AUC of 0.985 ± 0.002, accuracy of 0.948 ± 0.009, precision of 0.949 ± 0.009, recall of 0.968 ± 0.009, and F1 score of 0.959 ± 0.007. Similarly, the top 10 feature variables of GB model were Glasgow Coma Scale (GCS) score, vasoactive drugs, acute kidney failure, AIMS65 score, APACHE-II score, mechanical ventilation, the minimum of lactate, chronic liver disease, and the minimum and maximum of APTT. The SHAP visualization shows the weights of two ML models feature variables and the average sharp values of variables. Meanwhile, SHAP waterfall outputs the model prediction process with true positive and negative patients. Most importantly, two website prognostic prediction platforms were developed to enhance clinical accessibility: the ET model for AVGIB patients available at https://10zr656do5281.vicp.fun while the GB model for ANGIB patients accessible at http://10zr656do5281.vicp.fun.

**Conclusion:**

The ET model provides a reliable prognostic tool for AVGIB patients, while the GB model serves as a robust tool for ANGIB patients in predicting in-hospital mortality. By systematically integrating clinical features, risk stratification scores, vital signs, and invention measures, the ML models may deliver comprehensive predictions that benefit for clinical decision-making and potentially enhance clinical outcomes in the near future.

## 1 Introduction

Acute upper gastrointestinal bleeding (AUGIB) is a life-threatening disease frequently manifests as hemorrhagic shock, hypotension, multiple organ dysfunction (MODS), and even circulatory failure, with a mortality ranging from 15 to 20% (1). Approximately one-third of AUGIB patients require intensive care unit (ICU) admission, such as central venous catheterization, repaid liquid resuscitation, emergency tracheal intubation, endoscopy, and even vasoactive drugs (2). Due to the increasing aging population, numerous cardiovascular comorbidities, and the unhealthy lifestyles, the incidence of AUGIB patients has been rising significantly. According to the 2021 American College of Gastroenterology (ACG) guidelines, the estimated incidence ranges from 100 to 180 per 100,000 individuals, with mortality between 10 and 15% (3).

Risk stratification is the key priority in AUGIB management, as emphasized by major international guidelines (3–5). Previously, existing scoring systems such as AIMS65 (6), Glasgow-Blatchford score (GBS) (7), and Rockall score (8) have been used for risk prediction and stratification but exhibit limited sensitivity and specificity in forecasting mortality, rebleeding, and the need for therapeutic intervention. The ICU physicians are unfamiliar with scores other than APACHE-II and GCS scores, which might not be optimized for AUGIB patients (9). The heterogeneity of AUGIB patients, with varying in ages, genders, etiologies, bleeding sites, and blood loss volumes, further complicates accurate outcome prediction. Previous studies on gastrointestinal bleeding have primarily focused on the emergency departments (10) and gastroenterology departments (11). The Ungureanu ML model is restricted to patients with non-variceal upper gastrointestinal bleeding (12). Given the high mortality risk associated with AUGIB, the majority of patients required ICU admission for more attention.

The advent of artificial intelligence (AI) has transformed medical practice, particular in precision diagnosis, personalized therapy strategies, and prognostic prediction (13). Thus, we sought to develop dedicated ML models for mortality prediction for in ICU admitted AUGIB patients, incorporating with comprehensive variables. Recognizing the critical importance of disease stratification, we distinguished AUGIB into variceal and non-variceal subtypes, as these conditions demonstrate substantial differences in both clinical management and prognosis outcomes (14). To identify optimal predictive models, we utilized the Medical Information Mart for Intensive Care (MIMIC)-IV, comprehensive critical care database from the United States. Our methodology involved evaluating 12 machine learning algorithms and implementing various data imbalance techniques to enhance the model’s performance. The rigorous approach enabled us to develop two distinct prediction models, one for variceal and another for non-variceal AUGIB patients. Ultimately, two separate prediction models offer improved comprehensive, precision, and highly accuracy, representing significant advancements in mortality risk stratification for high-risk patient population.

## 2 Materials and methods

### 2.1 Data resource and ethical issues

The MIMIC-IV database represents an open-access critical care repository developed and maintained by the Massachusetts Institute of Technology (MIT) laboratory for computational physiology. It provides longitudinal clinical records for inpatients at the Boston-based Beth Israel Deaconess Medical Center spanning 2008 to 2019 (15). It encompasses a wide array of clinical parameters including demographics, continuous vital signs measurements, laboratory results, diagnostic codes, medication administration records, therapeutic interventions, and additional clinically relevant data.

Access to database was obtained through proper institutional channels, with authorized certification No. 12760266. All patient’s private information was anonymized to meet ethical regulations, and the study was granted exemption from requiring informed consent by the institution review board.

### 2.2 Inclusion and exclusion criteria

Patients were included based on the following criteria: (1) Diagnosis of AUGIB according to the American Gastroenterological Association (AGA) guidelines was enrolled (3). (2) Documented ICD codes confirming upper gastrointestinal etiology, including but not limited to esophageal varices with bleeding (4560), peptic ulcer hemorrhage (K25.0, K26.0, et al.), acute hemorrhagic gastritis (K29.01), Mallory-Weiss syndrome (K22.6), acute gastrojejunal ulcer with hemorrhage (K280), and angiodysplasia of stomach and duodenum with bleeding (K31.811).

Patients were excluded if they met any of the following criteria: (1) age under 18 years old. (2) ICU hospitalization duration less than 24 h; (3) primary diagnosis of lower gastrointestinal bleeding; (4) incomplete clinical records, defined as more than 20% missing values. (5) pregnancy of postpartum status.

### 2.3 Data extraction and processing

The study utilized data from MIMIC-IV database, which was obtained through authorized access from PhysioNet. Get the access of downloaded, and subsequently installed and imported into PostGres 12.0 software. All data retrieval and extraction were performed by Structured Query Language (SQL) to ensure precision and reproducibility.

Clinical risk scores, including the AIMS65 score, Rockall score, shock index, and GBS score, were calculated based on variables extracted from each patient’s initial medical record. Due to continuous monitoring and multiple dynamic follow-ups, we captured patient’s vital signs and laboratory parameters during the first 24 h of ICU admission: the minimum, the maximum, and average values. Acute variceal gastrointestinal bleeding (AVGIB) and acute non-variceal gastrointestinal bleeding (ANGIB) patients’ demographics, vital signs, laboratory results, medications, diagnoses, interventions, and other clinical data were included as possible related variables.

### 2.4 Machine learning models

The comprehensive analysis employed 12 distinct machine learning algorithms, each selected for unique strengths in predictive model.

(1) Logistic Regression (LR) A fundamental classification algorithms particularly effective for binary problems, LR provides interpretable results by quantifying the contribution of each independent variable through odds ratios (16).
(2) Decision Trees (DT) The intuitive algorithm utilizes hierarchical tree structure to model decision pathways, capable of capturing non-linear relationships without requiring feature scaling. The DT visual interpretability makes it particularly valuable in medical applications (17).
(3) Random Forest (RF) The ensemble learning method that constructs multiple decision trees during the training. The RF aggregates predictions through either majority voting (classification) or averaging (regression), significantly improving prediction stability (18).
(4) Gradient Boosting (GBoost) It involves a sequence of weak models (such as decision trees) in specific order to minimize given loss function, which automatically handle missing values, offering high accuracy and versatility across diverse tasks (19).
(5) Adaptive Boosting (AdaBoost) The ensemble algorithm that specifically targets misclassification by adjusting the weights of misclassified instances and iteratively refining the model (20).
(6) eXtreme Gradient Boosting (XGBoost) The optimized distributed gradient boosting algorithms designed to be highly efficient, flexible, and portable, XGBoost minimizes the objective function through iterative training and perform regularization to prevent overfitting (21).
(7) Naive Bayes (NB) The probabilistic algorithm based on Bayes’ theorem, assuming feature independence, is particularly effective for multiple classification tasks and lower dimensional data (22).
(8) Support Vector Machine (SVM) The SVM with RBF kernel uses the “kernel trick” maps to implicitly features into a higher-dimensional space for non-linear decision bound with high sensitivity (23).
(9) Light Gradient-Boosting Machine (LightGBM) The gradient boosting framework using tree-based learning algorithms could hand large datasets and categorical features effectively with leaf-wise growth strategy (24).
(10) K-Nearest Neighbors (KNN) The simple and instance-based learning algorithm that classifies a data point based on the majority vote of its neighbors, where the distance metric significantly impacts performance and requires normalization for consistent results (25).
(11) Extra Trees (ET) The ensemble algorithm by creating multiple decision trees through randomizing feature splits and dataset sampling, which not only reduced variance but also improved model robustness (26).
(12) Voting Classifier (VC) The ensemble of multiple models that reduces the bias and standard deviation of individual models, resulting in a more robust and reliable performance (27).

### 2.5 Model evaluation

To effectively mitigate the challenges posed by dataset imbalance, we implemented two advanced resampling techniques, namely, Edited Nearest Neighbors (SMOTE-ENN) technique and Adaptive Synthetic Sampling (ADASYN) technique to enhance model accuracy (28, 29). For robust model validation, we employed dual validation strategy: 5-fold cross-validation (CV) and independent validation (IV) to minimize overfitting risk (30).

The model evaluation incorporated multiple complementary metrics: classification metrics and discriminatory performance. The key classification metrics contained accuracy, precision, recall, and F1-score. The critical parameter of discriminatory performance included receiver operating characteristic (ROC) curve analysis. The area under the curve (AUC) analysis was conducted to evaluate the predictive performance among different models.

### 2.6 Model explainability

The SHAP algorithm was used to analyze the importance of features and present the contribution of feature variables based on ML model predictions to explain the model’s prediction process (31). To enhance model interpretability and trustworthiness, summary and waterfall plots were constructed to understand the decision-making process better. The ML models randomly generated positive and negative analysis of prediction process.

### 2.7 Statistical analysis

Descriptive statistics were presented as means with standard deviations (SDs), medians with interquartile ranges, or counts with frequencies, depending on the data type and distribution. Comparisons between groups were conducted using Fisher’s exact test for categorical data and the *t*-test, Wilcoxon rank-sum test, analysis of variance, or Kruskal–Wallis test for continuous data as appropriate. Patients were divided into two groups according to the in-hospital outcome. The ML models ultimately included variables with *p*-value less than 0.001. Python programming software (version 3.9) was used in data processing and model evaluation.

## 3 Results

### 3.1 Study research design process

**Figure 1:**
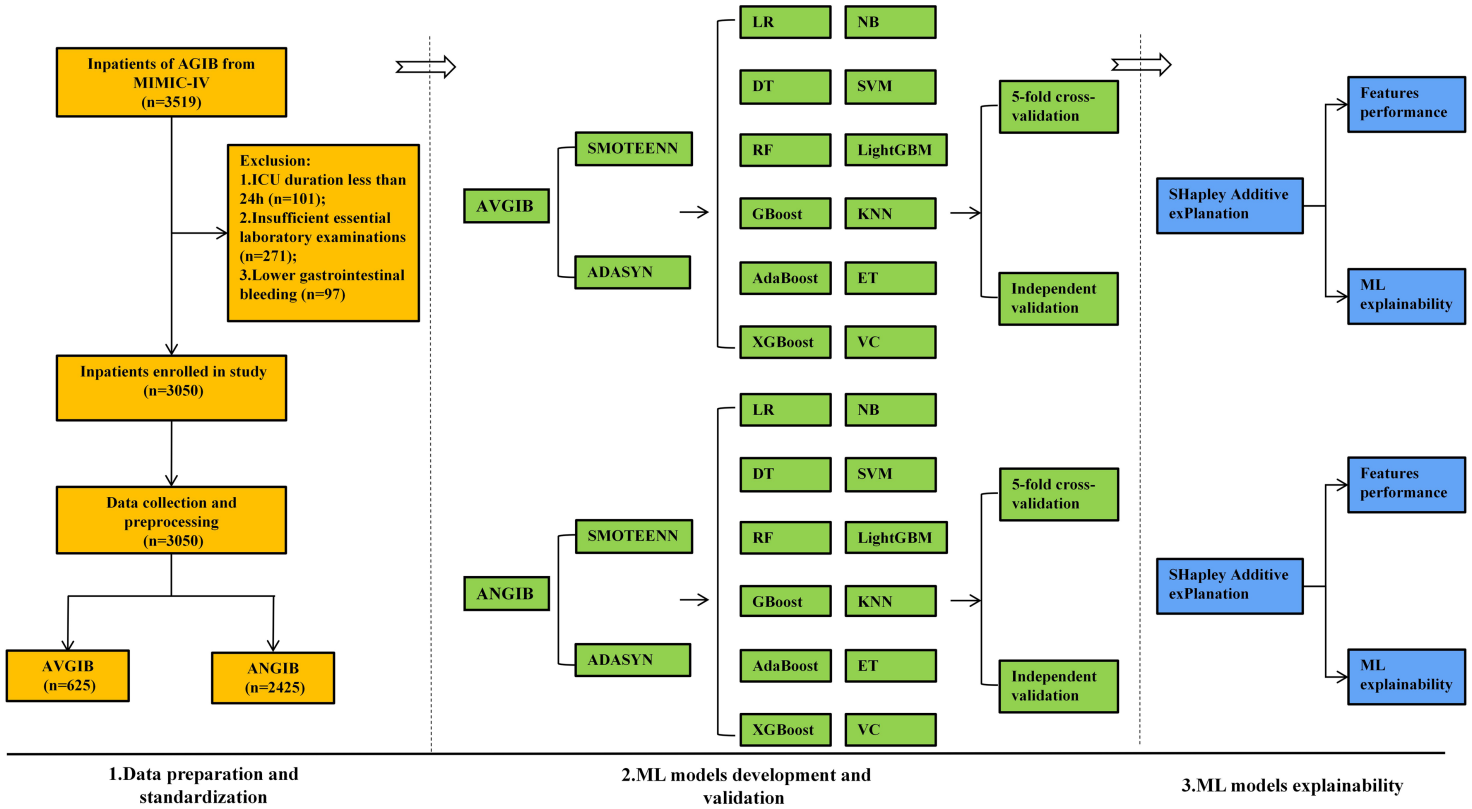
Technical roadmap for constructing machine learning model. SMOTE-ENN: Synthetic Minority Over-sampling Technique-Edited Nearest Neighbors; ADASYN: Adaptive Synthetic Sampling; LR: logistic regression; DT: decision tree; RF: random forest; GBoost: gradient boosting; AdaBoost: adaptive boosting; XGBoost: eXtreme Gradient Boosting; NB: Naive Bayes; SVM: support vector machine; LightGBM: light gradient-boosting machine; KNN: K-nearest neighbors; ET: extra trees; VC: voting classifier.

The prediction model construction comprises three core components, namely, data preprocessing and standardization, ML models development and validation, models explainability analysis (Figure 1). Initial screening of the MIMIC-IV database identified 3,519 patients, 101 patients were excluded due to less than 24 h ICU duration, 97 patients were excluded for lower gastrointestinal bleeding, and 271 patients were removed for insufficient data more than 20% missing. Ultimately, a total of 3,050 patients were included in the cohort, 625 patients (20.5%) were stratified into AVGIB, and the remaining 2,425 patients (79.5%) were defined as ANGIB, described in Supplementary Table. The cohort of 3,050 AUGIB patients demonstrated an overall mortality rate of 19.15% (*n* = 584), aligning with established epidemiological data from prior studies (32). The study included 3,050 AUGIB patients with numerous variables, including demographic characteristics, medication history, special interventions, vital signs, medical history, blood transfusions, severity scores, and laboratory results (Table 1).

**Table 1:** Clinical characteristics of AUGIB patients in MIMIC-IV database.

No.	Feature variable	Overall (*n* = 3,050)
Demographics		
1	Age, y	64.81 (54.07, 77.31)
2	Male, *n* (%)	1883 (61.74)
Medical history		
3	Anticoagulants, *n* (%)	507 (16.62)
4	Antiplatelet agents, *n* (%)	1,002 (32.85)
5	Proton pump inhibitors, *n* (%)	2,941 (96.43)
Intervention measures		
6	Vasoactive drugs, *n* (%)	1,030 (33.77)
7	CRRT, *n* (%)	209 (6.85)
8	Mechanical ventilation, *n* (%)	1,319 (43.25)
Vital signs		
9	Heart rate_min, bmp	72.00 (63.00, 84.00)
10	Heart rate_max, bmp	105.00 (91.00, 119.00)
11	Heart rate_mean, bmp	86.88 (75.93, 98.21)
12	Respiratory rate_min, bmp	12.00 (10.00, 15.00)
13	Respiratory rate_max, bmp	27.00 (24.00, 31.00)
14	Respiratory rate_mean, bmp	18.59 (16.43, 21.33)
15	SPB_min, mmHg	90.00 (80.00, 100.00)
16	SPB_max, mmHg	143.00 (128.00,160.00)
17	SPB_mean, mmHg	113.74 (104.07,126.71)
18	DBP_min, mmHg	45.00 (38.00, 53.00)
19	DBP_max, mmHg	86.00 (75.00, 99.00)
20	DBP_mean, mmHg	61.48 (54.70, 69.67)
21	MBP_min, mmHg	58.00 (50.00, 65.00)
22	MBP_max, mmHg	100.00 (88.00, 113.00)
23	MBP_mean, mmHg	75.28 (68.80, 83.39)
24	SpO_2__min, %	93.00 (90.00, 95.00)
25	SpO_2__max, %	100.00 (100.00,100.00)
26	SpO_2__mean, %	97.42 (96.08, 98.65)
Previous history		
27	Myocardial infarction, *n* (%)	472 (15.48)
28	Congestive heart failure, *n* (%)	911 (29.87)
29	Hypertension, *n* (%)	803 (26.33)
30	Diabetes, *n* (%)	973 (31.90)
31	Atrial fibrillation, *n* (%)	788 (25.84)
32	Chronic kidney disease, *n* (%)	851 (27.90)
33	COPD, *n* (%)	321 (10.52)
34	Chronic liver disease, *n* (%)	1,350 (44.26)
Blood transfusion		
35	Red blood cells, *n* (%)	1912 (62.69)
36	Plasma, *n* (%)	752 (24.66)
37	Platelets, *n* (%)	548 (17.97)
38	Albumin, *n* (%)	612 (20.1)
Related scores		
39	APACHE-II	12.00 (9.00, 16.00)
40	GBS	13.00 (10.00, 15.00)
41	AIMS65	2.00 (1.00, 3.00)
42	Rockall score	7.00 (6.00, 7.00)
43	Shock index	0.76 (0.63, 0.92)
44	GCS score	13.00 (10.00, 15.00)
Laboratory Result		
45	WBC_min, 10^9^/L	8.20 (5.50, 12.00)
46	WBC_max, 10^9^/L	11.70 (7.80, 17.10)
47	WBC_mean, 10^9^/L	10.02 (6.73, 14.35)
48	RBC_min, 10^12^/L	2.74 (2.35, 3.22)
49	RBC_max, 10^12^/L	3.28 (2.90, 3.77)
50	RBC_mean, 10^12^/L	3.00 (2.67, 3.47)
51	Hb_min, mg/dL	8.20 (7.00, 9.70)
52	Hb_max, mg/dL	9.90 (8.70, 11.30)
53	Hb_mean, mg/dL	9.03 (7.97, 10.37)
54	PLT_min, 10^9^/L	134.00 (76.00, 210.00)
55	PLT_max, 10^9^/L	180.00 (111.00, 265.00)
56	PLT_mean, 10^9^/L	155.63 (94.07, 231.67)
57	ALT_min,U/L	23.00 (14.00, 42.00)
58	ALT_max,U/L	25.00 (15.00, 50.00)
59	ALT_mean,U/L	24.00 (15.00, 46.94)
60	AST_min,U/L	35.00 (21.00, 77.00)
61	AST_max,U/L	40.00 (22.00, 91.00)
62	AST_mean,U/L	38.00 (21.75, 84.95)
63	Albumin_min, mg/dL	3.00 (2.60, 3.43)
64	Albumin_max, mg/dL	3.10 (2.67, 3.50)
65	Albumin_mean, mg/dL	3.05 (2.60, 3.50)
66	TBIL_min, mg/dL	0.92 (0.50, 2.44)
67	TBIL_max, mg/dL	1.10 (0.53, 3.00)
68	TBIL_mean, mg/dL	1.05 (0.50, 2.79)
69	BUN_min, mg/dL	25.00 (15.00, 42.00)
70	BUN_max, mg/dL	30.00 (18.00, 52.00)
71	BUN_mean, mg/dL	27.50 (17.00, 47.33)
72	Creatinine_min, mg/dL	1.00 (0.70, 1.70)
73	Creatinine_max, mg/dL	1.20 (0.80, 2.10)
74	Creatinine_mean, mg/dL	1.12 (0.77, 1.90)
75	Lactate_min, mmol/L	1.60 (1.10, 2.30)
76	Lactate_max, mmol/L	1.60 (1.20, 2.45)
77	Lactate_mean, mmol/L	1.60 (1.10, 2.50)
78	Potassium_min, mmol/L	3.90 (3.50, 4.30)
79	Potassium_max, mmol/L	4.40 (4.00, 5.00)
80	Potassium_mean, mmol/L	4.15 (3.80, 4.60)
81	Sodium_min,mmol/L	137.00 (134.00, 140.00)
82	Sodium_max,mmol/L	140.00 (137.00, 143.00)
83	Sodium_mean,mmol/L	138.78 (135.50, 141.50)
84	PT_min, sec	14.60 (12.80, 18.10)
85	PT_max, sec	16.00 (13.40, 21.20)
86	PT_mean, sec	15.40 (13.17, 19.69)
87	APTT_min, sec	30.20 (26.40, 36.00)
88	APTT_max, sec	33.90 (28.60, 46.20)
89	APTT_mean, sec	32.42 (27.84, 41.06)
90	INR_min	1.30 (1.20, 1.70)
91	INR_max	1.45 (1.20, 2.00)
92	INR_mean	1.40 (1.20, 1.80)

The ML models were separately developed according to the AVGIB and ANGIB subgroups. To address data imbalance and enhance model generalizability, we employed advanced data balancing techniques including SMOTE-ENN and ADASYN. Twelve machine learning algorithms were systematically evaluated through 5-fold cross validation and independent validation. The optimal models were selected based on comprehensive performance metrics including AUC, accuracy, precision, recall, and F1-score.

The Shapley Additive exPlanations (SHAP) algorithms were introduced to identify the top 20 most influential features through SHAP summary plots, mean SHAP value analysis, and visualize prediction mechanism via decision plots for representative cases and force plots illustrating positive and negative predictions.

### 3.2 Acute variceal gastrointestinal bleeding patients machine learning model

#### 3.2.1 Clinical characteristic and predictor screening

The study identified 625 patients with acute variceal gastrointestinal bleeding through ICD code verification. Based on clinical outcomes, 497 patients (79.5%) were stratified into survival group and 128 patients (20.5%) were classified into non-survival group. The clinical characteristic of demographics, medical history, previous history, intervention measures, vital sign, related scores, and laboratory results were collected.

The variceal bleeding study initially evaluated 92 candidate variables, with 59 demonstrating statistically significant associations (*p* < 0.001) upon rigorous screening. The feature variable selection results are presented in Table 2. To enhance the sensitivity of the ML model, we only incorporated 59 variables with *p* < 0.001.

**Table 2:** Feature characteristics of AVGIB patients in survival and non-survival groups.

Variables	Total (*n* = 625)	Survival (*n* = 497)	Non-survival (*n* = 128)	*p*-value
Demographics				
Age, y	57.08 (47.63, 63.58)	57.46 (48.06, 63.37)	53.54 (45.70, 64.51)	0.211
Male, n(%)	444 (71.04%)	353 (71.03%)	91 (71.09%)	0.999
Medical history				
Anticoagulants, n(%)	19 (3.04%)	17 (3.42%)	2 (1.56%)	0.422
Antiplatelet agents, n(%)	60 (9.60%)	49 (9.86%)	11 (8.59%)	0.791
Proton pump inhibitors, n(%)	624 (99.84%)	497 (100.00%)	127 (99.22%)	0.464
Intervention measures				
Vasoactive drugs, n(%)	214 (34.24%)	126 (25.35%)	88 (68.75%)	<0.001
CRRT, n(%)	40 (6.40%)	12 (2.41%)	28 (21.88%)	<0.001
Mechanical ventilation, n(%)	375 (60.00%)	276 (55.53%)	99 (77.34%)	<0.001
Vital sign				
Heart rate_min, bmp	72.00 (63.00, 84.00)	71.00 (62.00, 81.00)	78.00 (65.75, 89.00)	<0.001
Heart rate_max, bmp	105.00 (90.00, 117.00)	103.00 (89.00, 116.00)	110.00 (97.50, 120.00)	<0.001
Heart rate_mean, bmp	86.82 (75.29, 97.43)	84.88 (74.12, 96.26)	92.24 (79.23, 103.64)	<0.001
Respiratory rate_min, bmp	12.00 (9.00, 14.00)	11.00 (9.00, 14.00)	13.00 (9.75, 15.00)	0.006
Respiratory rate_max, bmp	26.00 (22.50, 30.00)	25.00 (22.00, 30.00)	28.00 (23.00, 32.00)	0.007
Respiratory rate mean, bmp	17.61 (15.48, 20.03)	17.35 (15.35, 19.44)	19.19 (16.34, 23.14)	<0.001
SBP_min, mmHg	89.00 (81.00, 99.00)	91.00 (82.00, 101.00)	83.00 (75.00, 91.25)	<0.001
SBP_max, mmHg	141.00 (126.00, 156.00)	143.00 (129.00, 157.00)	132.00 (120.00, 146.00)	<0.001
SBP_mean, mmHg	111.97 (103.00, 122.82)	114.04 (105.17, 124.54)	103.78 (97.10, 111.74)	<0.001
DBP_min, mmHg	46.00 (40.00, 54.00)	47.00 (41.00, 55.00)	43.00 (37.00, 49.25)	<0.001
DBP_max, mmHg	86.00 (76.00, 97.00)	87.00 (77.00, 97.00)	81.00 (70.00, 92.25)	<0.001
DBP_mean, mmHg	62.84 (56.78, 70.00)	64.00 (57.60, 70.90)	59.35 (53.22, 64.09)	<0.001
MBP_min, mmHg	58.00 (51.00, 66.00)	59.00 (53.00, 67.00)	54.00 (48.00, 60.00)	<0.001
MBP_max, mmHg	98.00 (89.00, 111.00)	100.00 (90.00, 112.00)	94.00 (85.00, 109.00)	0.015
MBP_mean, mmHg	75.78 (69.40, 82.81)	77.00 (70.30, 83.93)	70.83 (66.16, 77.18)	<0.001
SpO_2__min, %	93.00 (91.00, 96.00)	93.00 (92.00, 96.00)	93.00 (90.00, 96.00)	0.029
SpO_2__max, %	100.00 (100.00, 100.00)	100.00 (100.00, 100.00)	100.00 (100.00, 100.00)	0.594
SpO_2__mean, %	97.53 (96.36, 98.79)	97.58 (96.47, 98.79)	97.43 (95.87, 98.83)	0.253
Previous history				
Myocardial infarction, *n* (%)	30 (4.80%)	11 (2.21%)	19 (14.84%)	<0.001
Congestive heart failure, *n* (%)	53 (8.48%)	35 (7.04%)	18 (14.06%)	0.018
Hypertension, *n* (%)	71 (11.36%)	67 (13.48%)	4 (3.12%)	0.002
Diabetes, *n* (%)	158 (25.28%)	143 (28.77%)	15 (11.72%)	<0.001
Atrial fibrillation, *n* (%)	46 (7.36%)	35 (7.04%)	11 (8.59%)	0.682
Chronic kidney disease, *n* (%)	73 (11.68%)	55 (11.07%)	18 (14.06%)	0.431
COPD, *n* (%)	32 (5.12%)	24 (4.83%)	8 (6.25%)	0.670
Chronic liver disease, *n* (%)	584 (93.44%)	458 (92.15%)	126 (98.44%)	0.018
Blood transfusion				
i_RBC	413 (66.08%)	314 (63.18%)	99 (77.34%)	0.004
i_FFP	233 (37.28%)	142 (28.57%)	91 (71.09%)	<0.001
i_PLT	173 (27.68%)	105 (21.13%)	68 (53.12%)	<0.001
i_ALB	213 (34.08%)	137 (27.57%)	76 (59.38%)	<0.001
Related scores				
APACHE-II	10.00 (8.00, 15.00)	10.00 (8.00, 13.00)	15.00 (11.00, 19.00)	<0.001
GBS	13.00 (10.00, 15.00)	13.00 (10.00, 15.00)	14.00 (10.75, 15.00)	<0.001
AIMS65	2.00 (1.00, 3.00)	2.00 (1.00, 2.00)	3.00 (2.00, 3.00)	<0.001
Rockall	7.00 (6.00, 7.00)	7.00 (6.00, 7.00)	7.00 (6.00, 8.00)	0.034
Shock Index	2.00 (1.00, 3.00)	2.00 (1.00, 2.00)	3.00 (2.00, 3.00)	<0.001
GCS	13.00 (10.00, 15.00)	13.00 (10.00, 15.00)	14.00 (10.75, 15.00)	<0.001
Laboratory results				
WBC_min, 10^9^/L	6.90 (4.20, 10.40)	6.60 (4.00, 9.50)	9.00 (5.90, 14.50)	<0.001
WBC_max, 10^9^/L	10.20 (6.70, 15.90)	9.30 (6.30, 14.00)	14.15 (10.00, 22.00)	<0.001
WBC_mean, 10^9^/L	8.45 (5.47, 12.70)	7.80 (5.20, 11.38)	11.89 (7.47, 18.03)	<0.001
RBC_min, 10^12^/L	2.68 (2.30, 3.10)	2.77 (2.36, 3.16)	2.50 (2.08, 2.75)	<0.001
RBC_max, 10^12^/L	3.25 (2.89, 3.67)	3.27 (2.92, 3.71)	3.14 (2.76, 3.43)	0.002
RBC_mean, 10^12^/L	2.98 (2.64, 3.35)	3.05 (2.67, 3.41)	2.82 (2.51, 3.03)	<0.001
Hemoglobin_min, mg/dL	8.30 (7.20, 9.60)	8.40 (7.30, 9.70)	7.80 (6.70, 8.93)	<0.001
Hemoglobin_max, mg/dL	10.00 (8.80, 11.20)	10.00 (8.80, 11.20)	10.00 (8.88, 11.03)	0.929
Hemoglobin_mean, mg/dL	9.15 (8.05, 10.36)	9.20 (8.06, 10.40)	8.91 (8.05, 10.00)	0.077
Platelets_min, 10^9^/L	77.00 (50.00, 120.00)	80.00 (51.00, 127.00)	67.00 (45.00, 104.25)	0.004
Platelets_max, 10^9^/L	116.00 (77.00, 168.00)	116.00 (78.00, 174.00)	113.00 (76.75, 154.00)	0.218
Platelets_mean, 10^9^/L	96.00 (64.75, 140.50)	97.50 (65.00, 149.00)	85.50 (63.38, 118.05)	0.058
ALT_min, U/L	30.00 (19.00, 51.00)	28.00 (18.00, 45.00)	37.50 (23.00, 76.25)	<0.001
ALT_max, U/L	35.00 (22.00, 63.00)	32.00 (21.00, 55.00)	45.50 (26.00, 114.50)	<0.001
ALT_mean, U/L	32.50 (21.00, 57.00)	31.00 (20.00, 51.50)	41.50 (24.75, 97.67)	<0.001
AST_min, U/L	57.00 (35.00, 111.00)	54.00 (33.00, 96.00)	88.50 (38.75, 177.75)	<0.001
AST_max, U/L	68.00 (40.00, 140.00)	65.00 (40.00, 125.00)	106.00 (44.00, 295.50)	<0.001
AST_mean, U/L	62.50 (38.00, 126.00)	59.00 (37.50, 113.00)	101.25 (39.88, 231.88)	<0.001
ALB_min, mg/dL	2.99 ± 0.62	3.02 ± 0.60	2.87 ± 0.70	0.019
ALB_max, mg/dL	3.10 (2.70, 3.50)	3.13 (2.70, 3.50)	2.90 (2.50, 3.46)	0.027
ALB_mean, mg/dL	3.00 (2.62, 3.41)	3.05 (2.70, 3.46)	2.88 (2.50, 3.40)	0.014
TBIL_min, mg/dL	2.40 (1.10, 6.00)	1.90 (0.90, 4.10)	7.40 (3.20, 16.82)	<0.001
TBIL_max, mg/dL	3.10 (1.40, 7.40)	2.40 (1.20, 5.00)	8.50 (4.30, 20.38)	<0.001
TBIL_mean, mg/dL	2.80 (1.25, 6.75)	2.10 (1.10, 4.55)	7.86 (4.04, 17.75)	<0.001
BUN_min, mg/dL	24.00 (15.00, 40.00)	22.00 (14.00, 36.00)	36.50 (23.00, 50.00)	<0.001
BUN_max, mg/dL	29.00 (18.00, 47.00)	27.00 (17.00, 43.00)	42.00 (28.00, 60.75)	<0.001
BUN_mean, mg/dL	26.45 (16.52, 44.19)	24.50 (15.94, 38.50)	40.00 (25.40, 55.63)	<0.001
Creatinine_min, mg/dL	0.90 (0.60, 1.40)	0.80 (0.60, 1.20)	1.40 (0.90, 2.40)	<0.001
Creatinine_max, mg/dL	1.00 (0.80, 1.80)	0.90 (0.70, 1.40)	1.95 (1.00, 3.00)	<0.001
Creatinine_mean, mg/dL	0.95 (0.70, 1.62)	0.90 (0.69, 1.29)	1.70 (1.00, 2.59)	<0.001
Lactate_min, mmol/L	1.60 (1.30, 2.60)	1.60 (1.20, 2.30)	2.30 (1.60, 4.20)	<0.001
Lactate_max, mmol/L	1.60 (1.30, 2.70)	1.60 (1.20, 2.35)	2.35 (1.60, 4.10)	<0.001
Lactate_mean, mmol/L	1.60 (1.20, 2.70)	1.60 (1.10, 2.34)	2.00 (1.40, 3.95)	<0.001
Potassium_min, mmol/L	4.00 (3.60, 4.30)	4.00 (3.60, 4.30)	3.95 (3.58, 4.40)	0.994
Potassium_max, mmol/L	4.40 (4.00, 5.20)	4.40 (4.00, 5.10)	4.65 (4.00, 5.60)	0.076
Potassium_mean, mmol/L	4.20 (3.83, 4.70)	4.20 (3.83, 4.65)	4.25 (3.77, 5.08)	0.265
Sodium_min, mmol/L	137.00 (133.00, 140.00)	137.00 (134.00, 140.00)	135.00 (129.00, 141.00)	0.052
Sodium_max, mmol/L	140.00 (136.00, 143.00)	140.00 (137.00, 143.00)	138.00 (132.00, 145.00)	0.279
Sodium_mean, mmol/L	138.40 (135.00, 141.50)	138.50 (135.67, 141.00)	137.00 (131.38, 142.37)	0.126
PT_min, sec	16.70 (14.70, 19.70)	16.20 (14.10, 18.50)	20.00 (16.65, 23.25)	<0.001
PT_max, sec	18.70 (15.80, 23.40)	17.60 (15.20, 21.20)	24.65 (20.95, 32.77)	<0.001
PT_mean, sec	17.77 (15.50, 21.50)	16.90 (14.85, 19.87)	22.58 (18.85, 26.68)	<0.001
APTT_min, sec	32.70 (28.60, 38.10)	31.70 (28.10, 36.20)	39.35 (34.15, 46.28)	<0.001
APTT_max, sec	36.20 (31.20, 46.10)	34.30 (30.40, 41.50)	50.80 (38.72, 72.30)	<0.001
APTT_mean, sec	34.60 (30.22, 41.70)	33.15 (29.30, 38.51)	45.37 (36.27, 57.61)	<0.001
INR_min	1.50 (1.30, 1.80)	1.50 (1.30, 1.70)	1.85 (1.50, 2.20)	<0.001
INR_max	1.70 (1.40, 2.20)	1.60 (1.40, 2.00)	2.30 (1.90, 3.10)	<0.001
INR_mean	1.61 (1.40, 2.00)	1.55 (1.35, 1.85)	2.10 (1.73, 2.50)	<0.001

#### 3.2.2 Machine learning model construction and evaluation

Hybrid approaches combining SMOTE-ENN and ADASYN techniques were employed to simultaneously address data imbalances. Subsequently, 12 ML algorithms were adopted to select the optimal ML models. The dual validation strategy incorporating both 5-fold cross validation (Table 3) and independent validation (Table 4) serves as robust safeguard against overfitting by providing multiple performance estimates and maintaining completely unseen data for final evaluation.

**Table 3:** 5-fold cross-validation of ML models prediction performance for AVGIB patients.

Models	Mean AUC	Mean accuracy	Mean precision	Mean recall	Mean F1 score
ADASYN					
Logistic regression	0.852 ± 0.042	0.790 ± 0.032	0.491 ± 0.051	0.731 ± 0.080	0.586 ± 0.056
Decision tree	0.699 ± 0.013	0.760 ± 0.029	0.446 ± 0.044	0.641 ± 0.056	0.521 ± 0.013
Random forest	0.867 ± 0.047	0.826 ± 0.032	0.676 ± 0.202	0.373 ± 0.122	0.458 ± 0.106
Gradient boosting	0.843 ± 0.036	0.831 ± 0.030	0.634 ± 0.113	0.427 ± 0.085	0.505 ± 0.086
AdaBoost	0.847 ± 0.044	0.833 ± 0.038	0.662 ± 0.175	0.471 ± 0.051	0.539 ± 0.068
XGBoost	0.858 ± 0.057	0.840 ± 0.029	0.695 ± 0.159	0.439 ± 0.067	0.528 ± 0.069
Naive Bayes	0.850 ± 0.035	0.796 ± 0.031	0.513 ± 0.060	0.618 ± 0.097	0.552 ± 0.032
SVM (RBF Kernel)	0.874 ± 0.033	0.810 ± 0.045	0.541 ± 0.082	0.741 ± 0.104	0.616 ± 0.053
LightGBM	0.856 ± 0.030	0.831 ± 0.037	0.614 ± 0.112	0.575 ± 0.078	0.582 ± 0.050
KNN	0.784 ± 0.057	0.842 ± 0.031	0.800 ± 0.194	0.314 ± 0.065	0.449 ± 0.097
Extra trees	0.877 ± 0.030	0.842 ± 0.013	0.678 ± 0.073	0.461 ± 0.099	0.538 ± 0.057
Voting classifier	0.881 ± 0.038	0.847 ± 0.027	0.687 ± 0.130	0.529 ± 0.100	0.582 ± 0.063
SMOTE-ENN					
Logistic regression	0.993 ± 0.010	0.968 ± 0.007	0.965 ± 0.023	0.982 ± 0.018	0.973 ± 0.006
Decision tree	0.913 ± 0.026	0.929 ± 0.013	0.924 ± 0.019	0.957 ± 0.030	0.940 ± 0.011
Random forest	0.994 ± 0.008	0.956 ± 0.015	0.953 ± 0.020	0.972 ± 0.025	0.962 ± 0.013
Gradient boosting	0.982 ± 0.015	0.942 ± 0.024	0.940 ± 0.019	0.960 ± 0.023	0.950 ± 0.021
AdaBoost	0.990 ± 0.017	0.970 ± 0.021	0.973 ± 0.018	0.975 ± 0.029	0.974 ± 0.018
XGBoost	0.992 ± 0.012	0.970 ± 0.023	0.964 ± 0.020	0.985 ± 0.024	0.974 ± 0.020
Naive Bayes	0.953 ± 0.021	0.887 ± 0.028	0.938 ± 0.018	0.862 ± 0.063	0.897 ± 0.031
SVM (RBF Kernel)	0.995 ± 0.010	0.975 ± 0.014	0.962 ± 0.022	0.997 ± 0.006	0.979 ± 0.012
LightGBM	0.993 ± 0.011	0.973 ± 0.011	0.971 ± 0.020	0.985 ± 0.014	0.977 ± 0.009
KNN	0.983 ± 0.011	0.959 ± 0.023	0.936 ± 0.036	1.000 ± 0.000	0.966 ± 0.019
Extra trees	0.996 ± 0.007	0.966 ± 0.009	0.957 ± 0.024	0.988 ± 0.012	0.972 ± 0.007
Voting classifier	0.995 ± 0.008	0.970 ± 0.014	0.967 ± 0.019	0.982 ± 0.018	0.974 ± 0.012

**Table 4:** Independent validation of ML models prediction performance for AVGIB patients.

Models	Mean AUC	Mean accuracy	Mean precision	Mean recall	Mean F1 score
ADASYN					
Logistic regression	0.849 ± 0.031	0.805 ± 0.043	0.525 ± 0.074	0.738 ± 0.113	0.611 ± 0.083
Decision tree	0.692 ± 0.084	0.757 ± 0.027	0.440 ± 0.046	0.615 ± 0.081	0.512 ± 0.056
Random forest	0.852 ± 0.033	0.832 ± 0.018	0.642 ± 0.071	0.446 ± 0.062	0.524 ± 0.055
Gradient boosting	0.846 ± 0.018	0.837 ± 0.011	0.649 ± 0.051	0.485 ± 0.031	0.553 ± 0.016
AdaBoost	0.830 ± 0.033	0.818 ± 0.011	0.571 ± 0.024	0.485 ± 0.079	0.522 ± 0.053
XGBoost	0.841 ± 0.039	0.816 ± 0.015	0.566 ± 0.036	0.477 ± 0.086	0.515 ± 0.064
Naive Bayes	0.843 ± 0.034	0.798 ± 0.022	0.512 ± 0.039	0.685 ± 0.075	0.585 ± 0.048
SVM (RBF Kernel)	0.872 ± 0.027	0.814 ± 0.016	0.540 ± 0.031	0.738 ± 0.062	0.623 ± 0.035
LightGBM	0.845 ± 0.040	0.822 ± 0.015	0.572 ± 0.040	0.600 ± 0.062	0.583 ± 0.035
KNN	0.814 ± 0.040	0.824 ± 0.013	0.665 ± 0.072	0.315 ± 0.038	0.427 ± 0.044
Extra trees	0.871 ± 0.030	0.845 ± 0.015	0.658 ± 0.048	0.538 ± 0.049	0.590 ± 0.038
Voting classifier	0.865 ± 0.032	0.829 ± 0.015	0.598 ± 0.042	0.554 ± 0.039	0.574 ± 0.029
SMOTE-ENN					
Logistic regression	0.978 ± 0.007	0.941 ± 0.011	0.935 ± 0.017	0.966 ± 0.016	0.950 ± 0.009
Decision tree	0.912 ± 0.020	0.926 ± 0.014	0.926 ± 0.023	0.949 ± 0.012	0.937 ± 0.011
Random forest	0.994 ± 0.002	0.958 ± 0.014	0.945 ± 0.022	0.985 ± 0.009	0.965 ± 0.011
Gradient boosting	0.993 ± 0.003	0.960 ± 0.006	0.955 ± 0.018	0.979 ± 0.013	0.966 ± 0.005
AdaBoost	0.991 ± 0.004	0.958 ± 0.009	0.951 ± 0.017	0.979 ± 0.012	0.964 ± 0.008
XGBoost	0.992 ± 0.005	0.958 ± 0.006	0.949 ± 0.019	0.981 ± 0.012	0.964 ± 0.005
Naive Bayes	0.933 ± 0.021	0.852 ± 0.039	0.915 ± 0.024	0.821 ± 0.068	0.864 ± 0.041
SVM (RBF Kernel)	0.990 ± 0.006	0.962 ± 0.009	0.949 ± 0.011	0.987 ± 0.008	0.968 ± 0.008
LightGBM	0.993 ± 0.004	0.957 ± 0.007	0.955 ± 0.020	0.972 ± 0.016	0.963 ± 0.005
KNN	0.990 ± 0.007	0.956 ± 0.013	0.933 ± 0.021	0.996 ± 0.009	0.963 ± 0.011
Extra trees	0.996 ± 0.001	0.964 ± 0.007	0.951 ± 0.009	0.989 ± 0.007	0.970 ± 0.006
Voting classifier	0.994 ± 0.003	0.953 ± 0.006	0.940 ± 0.018	0.983 ± 0.011	0.961 ± 0.005

Through comprehensive evaluation of key parameters (including accuracy, precision, recall, and F1-score), the ET model exhibited excellent prediction performance with AUC ranking the first with 0.996 ± 0.007, accuracy of 0.966 ± 0.009, precision of 0.957 ± 0.024, recall of 0.988 ± 0.012, and F1-score of 0.972 ± 0.007, respectively. Thus, ET model demonstrated superior performance and was consequently selected as the excellent predictive framework.

#### 3.2.3 Machine learning model performance

As represented in Table 3, the SMOTE-ENN imbalance-handling technique achieves superior performance across nearly all metrics. Consequently, SMOTE-ENN was finally selected as the optimal algorithm, outperforming the ADASYN technique. The consistency performance observed in both 5-CV and IV confirmed that SMOTE-ENN effectively mitigates class imbalance issue without generalizing overfitting, making it the most reliable resampling approach.

**Figure 2:**
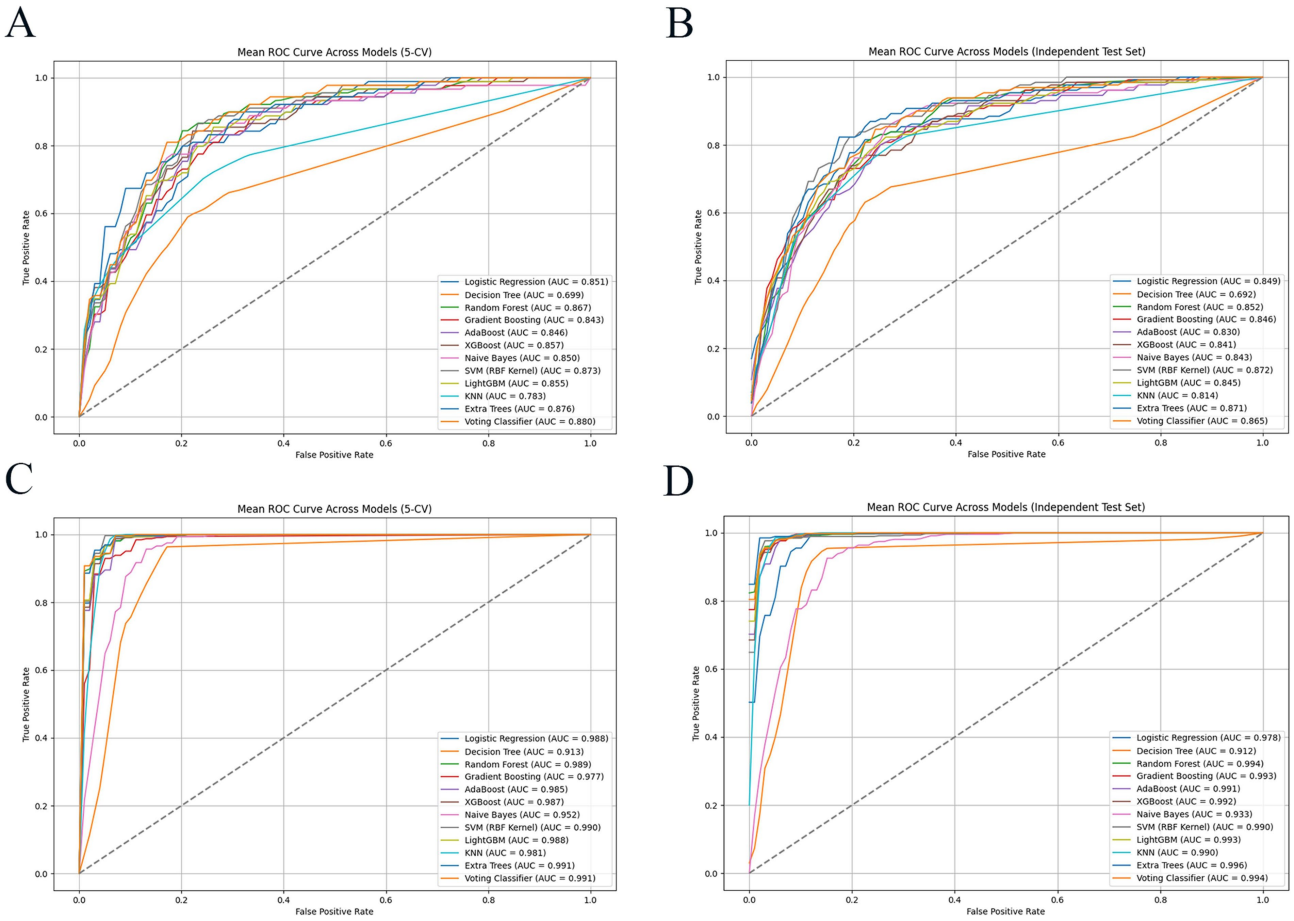
AUC of different machine learning models for AVGIB patients in 5 CV and IV. **(A)** The ROC of models by ADASYN technique in 5 CV; **(B)** the ROC of models by ADASYN technique in IV; **(C)** the ROC of models by SMOTE-ENN technique in 5 CV; **(D)** the ROC of models by SMOTE-ENN technique in IV. SMOTE-ENN: Synthetic Minority Over-sampling Technique-Edited Nearest Neighbors; ADASYN: Adaptive Synthetic Sampling.

Regarding the ML model performance, the AUC values varied significantly across different algorithms when using the ADASYN and SMOTE-ENN techniques, as evaluated through both 5-CV and IV in Figure 2. The ET model emerged as the optimal choice due to its highest AUC values and overall excellent performance, as detailed in Section 3.2.2.

#### 3.2.4 Machine learning model SHAP explainable

To directly display the weights of each feature variable and its predictive values of the ET model, SHAP (Shapley Additive exPlanations) algorithms were performed to visualize variables. The SHAP summary plot contained average SHAP values of variables and a representation of each feature’s contribution with SHAP values. The top 20 feature variables were acute kidney failure, transfusion albumin, vasoactive drugs, transfusion plasma, transfusion platelet, INR_max, PT_max, AIMS65 score, PT_mean, APTT_max, APACHE-II score, bilirubin_max, diabetes, INR_mean, bilirubin_mean, GCS score, APTT_min, INR_min, APTT_mean, and bilirubin_min.

**Figure 3:**
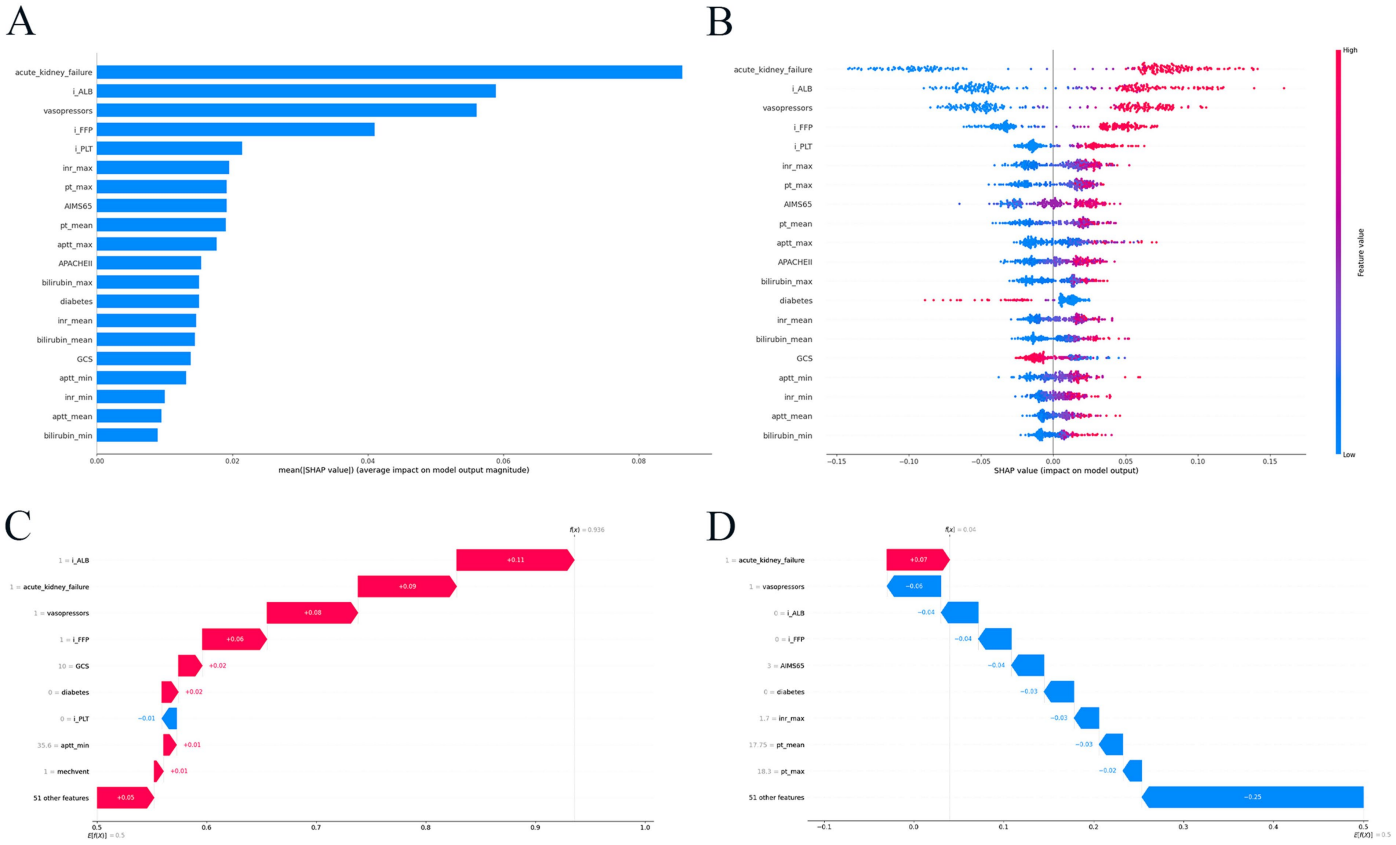
SHAP explainable of the extra trees prediction model. **(A)** The SHAP feature importance plot; **(B)** the SHAP summary plot; **(C)** the ML explainability of positive patient; **(D)** the ML explainability of negative patient.

The SHAP feature importance plot indicated top 20 feature variables weight of the optimal ET model in mortality prediction (Figure 3A). In the SHAP summary plot, each point represents the SHAP value of corresponding feature variable for given sample (Figure 3B). Similarly, points trending toward red color indicate higher feature values, while those approaching blue color denote lower feature values.

#### 3.2.5 Machine learning explainability for patients

The SHAP explains model predictions by quantifying feature contributions, visualized via waterfall plots. The positive patient was randomly selected in Figure 3C. The base value of the ET model is E *f*(*x*) = 0.05, the patient transfusion albumin, corresponding to *f*(*x*) = 0.11; patient had acute kidney failure, corresponding to *f*(*x*) = 0.09. Similarly, other feature variables correspond to *f*(*x*) values. As described in Figure 3C, the final *f*(*x*) was 0.936; therefore, the patient was positive case representative.

Similarly, the negative patient was randomly selected in Figure 3D. The base value of the ET model is E *f*(*x*) = −0.25, and the patient did not transfusion albumin, corresponding to *f*(*x*) = −0.04; patient had no diabetes, corresponding to *f*(*x*) = −0.03. Similarly, other feature variables correspond to *f*(*x*) values. As described in Figure 3D, the final *f*(*x*) was 0.04; therefore, the patient was negative case representative.

### 3.3 Acute non-variceal gastrointestinal bleeding patients machine learning model

#### 3.3.1 Clinical characteristic and predictor screening

The study identified 2,425 patients with acute non-variceal gastrointestinal bleeding through ICD code verification. Based on clinical outcomes, 1969 patients (81.2%) were stratified into survival group, and 456 patients (18.8%) were classified into non-survival group. The clinical characteristic of demographics, medical history, previous history, intervention measures, vital sign, related scores, and laboratory results are compared in Table 5.

**Table 5:** Feature characteristics of ANGIB patients in survival and non-survival groups.

Variables	Total (*n* = 2,425)	Survival (*n* = 1969)	Non-survival (*n* = 456)	*p*-value
Demographics				
Age, y	67.68 (56.38, 79.31)	67.52 (56.06, 79.37)	68.98 (57.59, 79.10)	0.211
Male, *n* (%)	1,439 (59.34%)	1,148 (58.30%)	291 (63.82%)	0.035
Medical History				
Anticoagulants, *n* (%)	488 (20.12%)	425 (21.58%)	63 (13.82%)	<0.001
Antiplatelet agents, *n* (%)	942 (38.85%)	781 (39.66%)	161 (35.31%)	0.096
PPI, *n* (%)	2,317 (95.55%)	1880 (95.48%)	437 (95.83%)	0.839
Intervention measures				
Vasoactive drugs, *n* (%)	816 (33.65%)	511 (25.95%)	305 (66.89%)	<0.001
CRRT, *n* (%)	169 (6.97%)	86 (4.37%)	83 (18.20%)	<0.001
Mechanical ventilation, *n* (%)	944 (38.93%)	641 (32.55%)	303 (66.45%)	<0.001
Vital sign				
Heart rate_min, bmp	72.00 (63.00, 84.00)	72.00 (63.00, 83.00)	76.00 (62.00, 88.00)	0.004
Heart rate_max, bmp	105.00 (92.00, 120.00)	104.00 (91.00, 119.00)	110.00 (97.00, 126.00)	<0.001
Heart rate_mean, bmp	86.96 (76.25, 98.46)	85.76 (75.43, 97.49)	91.11 (80.18, 103.68)	<0.001
Respiratory rate_min, bmp	12.00 (10.00, 15.00)	12.00 (10.00, 15.00)	13.00 (10.00, 16.00)	<0.001
Respiratory rate_max, bmp	27.00 (24.00, 32.00)	27.00 (24.00, 31.00)	29.00 (25.00, 34.00)	<0.001
Respiratory rate_mean, bmp	18.88 (16.69, 21.55)	18.65 (16.55, 21.14)	20.25 (17.38, 23.69)	<0.001
SBP_min, mmHg	90.00 (80.00, 101.00)	91.00 (82.00, 103.00)	83.00 (75.00, 92.00)	<0.001
SBP_max, mmHg	144.00 (129.00, 161.00)	145.00 (129.00, 162.00)	139.00 (123.00, 155.00)	<0.001
SBP_mean, mmHg	114.62 (104.41, 127.71)	116.20 (106.21, 129.30)	107.54 (100.11, 118.27)	<0.001
DBP_min, mmHg	44.00 (37.00, 52.00)	45.00 (38.00, 53.00)	41.00 (34.00, 49.00)	<0.001
DBP_max, mmHg	86.00 (74.00, 100.00)	87.00 (75.00, 100.00)	83.00 (70.00, 97.12)	<0.001
DBP_mean, mmHg	61.19 (54.15, 69.50)	62.04 (54.92, 70.25)	57.37 (51.08, 65.47)	<0.001
MBP_min, mmHg	57.00 (50.00, 65.00)	58.00 (51.00, 66.00)	53.00 (45.00, 61.00)	<0.001
MBP_max, mmHg	100.00 (88.00, 114.00)	100.00 (89.00, 114.00)	97.00 (86.00, 113.25)	0.002
MBP_mean, mmHg	75.15 (68.45, 83.50)	76.09 (69.47, 84.68)	71.31 (65.66, 78.39)	<0.001
SpO_2__min, %	93.00 (90.00, 95.00)	93.00 (91.00, 95.00)	92.00 (88.00, 94.00)	<0.001
SpO_2__max, %	100.00 (100.00, 100.00)	100.00 (100.00, 100.00)	100.00 (100.00, 100.00)	0.636
SpO_2__mean, %	97.41 (96.00, 98.63)	97.50 (96.20, 98.65)	96.95 (95.32, 98.56)	<0.001
Previous history				
Myocardial infarction, *n* (%)	442 (18.23%)	348 (17.67%)	94 (20.61%)	0.162
Congestive heart failure, *n* (%)	858 (35.38%)	667 (33.88%)	191 (41.89%)	0.002
Hypertension, *n* (%)	732 (30.19%)	617 (31.34%)	115 (25.22%)	0.012
Diabetes, *n* (%)	815 (33.61%)	664 (33.72%)	151 (33.11%)	0.847
Atrial fibrillation, *n* (%)	742 (30.60%)	580 (29.46%)	162 (35.53%)	0.013
Chronic kidney disease, *n* (%)	778 (32.08%)	617 (31.34%)	161 (35.31%)	0.114
COPD, *n* (%)	289 (11.92%)	219 (11.12%)	70 (15.35%)	0.015
Chronic liver disease, *n* (%)	766 (31.59%)	557 (28.29%)	209 (45.83%)	<0.001
Blood transfusion				
i_RBC	1,499 (61.81%)	1,203 (61.10%)	296 (64.91%)	0.145
i_FFP	519 (21.40%)	349 (17.72%)	170 (37.28%)	<0.001
i_PLT	375 (15.46%)	246 (12.49%)	129 (28.29%)	<0.001
i_ALB	399 (16.45%)	250 (12.70%)	149 (32.68%)	<0.001
Related scores				
APACHE-II	12.00 (10.00, 16.00)	12.00 (9.00, 15.00)	17.00 (12.00, 21.00)	<0.001
GBS	13.00 (10.00, 15.00)	13.00 (10.00, 15.00)	13.00 (10.00, 15.00)	0.003
AIMS65	2.00 (1.00, 3.00)	2.00 (1.00, 3.00)	3.00 (2.00, 3.00)	<0.001
Rockall	7.00 (5.00, 7.00)	6.00 (5.00, 7.00)	7.00 (6.00, 8.00)	<0.001
Shock Index	0.76 (0.63, 0.92)	0.75 (0.62, 0.90)	0.84 (0.69, 1.00)	<0.001
GCS	14.00 (11.00, 15.00)	14.00 (13.00, 15.00)	10.00 (6.00, 14.00)	<0.001
Laboratory results				
WBC_min, 10^9^/L	8.70 (5.90, 12.30)	8.30 (5.90, 11.70)	10.30 (6.38, 14.80)	<0.001
WBC_max, 10^9^/L	12.10 (8.20, 17.20)	11.70 (8.00, 16.40)	14.30 (9.60, 20.60)	<0.001
WBC_mean, 10^9^/L	10.42 (7.15, 14.60)	10.00 (7.00, 13.90)	12.20 (8.09, 17.61)	<0.001
RBC_min, 10^12^/L	2.75 (2.36, 3.27)	2.76 (2.38, 3.27)	2.71 (2.29, 3.24)	0.058
RBC_max, 10^12^/L	3.29 (2.90, 3.81)	3.31 (2.92, 3.81)	3.22 (2.85, 3.80)	0.030
RBC_mean, 10^12^/L	3.00 (2.67, 3.50)	3.03 (2.69, 3.51)	2.92 (2.59, 3.48)	0.023
Hemoglobin_min, mg/dL	8.20 (7.00, 9.70)	8.20 (7.00, 9.70)	8.20 (7.07, 9.80)	0.662
Hemoglobin_max, mg/dL	9.90 (8.60, 11.30)	9.90 (8.60, 11.30)	10.00 (8.60, 11.40)	0.591
Hemoglobin_mean, mg/dL	9.00 (7.96, 10.37)	9.01 (7.96, 10.35)	8.98 (7.96, 10.46)	0.634
Platelets_min, 10^9^/L	153.00 (96.00, 226.00)	158.00 (103.00, 230.00)	125.00 (61.00, 201.25)	<0.001
Platelets_max, 10^9^/L	199.00 (129.00, 286.00)	206.00 (138.00, 292.00)	167.50 (97.75, 258.25)	<0.001
Platelets_mean, 10^9^/L	176.00 (112.00, 250.00)	181.67 (119.50, 255.75)	145.67 (77.38, 223.12)	<0.001
ALT_min, U/L	21.00 (13.00, 40.00)	20.00 (13.00, 37.00)	27.00 (16.00, 59.00)	<0.001
ALT_max, U/L	23.00 (14.00, 45.00)	21.00 (13.00, 41.00)	31.00 (18.00, 72.00)	<0.001
ALT_mean, U/L	22.00 (13.50, 43.00)	21.00 (13.00, 39.00)	29.17 (17.00, 66.75)	<0.001
AST_min, U/L	30.00 (19.00, 65.00)	28.00 (19.00, 57.00)	49.00 (24.00, 104.50)	<0.001
AST_max, U/L	33.00 (20.00, 80.00)	31.00 (20.00, 66.00)	54.00 (26.00, 150.00)	<0.001
AST_mean, U/L	32.00 (20.00, 74.00)	30.00 (19.00, 62.00)	52.50 (25.19, 134.50)	<0.001
ALB_min, mg/dL	3.01 (2.60, 3.46)	3.09 (2.69, 3.50)	2.80 (2.30, 3.26)	<0.001
ALB_max, mg/dL	3.10 (2.66, 3.50)	3.10 (2.70, 3.59)	2.90 (2.40, 3.30)	<0.001
ALB_mean, mg/dL	3.05 (2.60, 3.50)	3.10 (2.70, 3.51)	2.85 (2.35, 3.30)	<0.001
TBIL_min, mg/dL	0.80 (0.40, 1.70)	0.70 (0.40, 1.47)	1.30 (0.50, 4.33)	<0.001
TBIL_max, mg/dL	0.90 (0.50, 2.20)	0.80 (0.50, 1.70)	1.60 (0.60, 5.93)	<0.001
TBIL_mean, mg/dL	0.80 (0.45, 1.95)	0.80 (0.45, 1.60)	1.48 (0.57, 5.26)	<0.001
BUN_min, mg/dL	25.00 (15.00, 43.00)	24.00 (15.00, 40.00)	32.00 (19.00, 56.00)	<0.001
BUN_max, mg/dL	31.00 (18.00, 52.00)	29.00 (18.00, 49.00)	39.00 (24.00, 65.00)	<0.001
BUN_mean, mg/dL	27.67 (17.00, 47.60)	26.33 (16.33, 45.00)	35.67 (21.50, 61.67)	<0.001
Creatinine_min, mg/dL	1.10 (0.70, 1.80)	1.00 (0.70, 1.70)	1.40 (0.80, 2.30)	<0.001
Creatinine_max, mg/dL	1.30 (0.90, 2.20)	1.20 (0.80, 2.00)	1.80 (1.10, 2.90)	<0.001
Creatinine_mean, mg/dL	1.19 (0.80, 2.00)	1.10 (0.77, 1.84)	1.60 (1.00, 2.55)	<0.001
Lactate_min, mmol/L	1.60 (1.10, 2.30)	1.60 (1.00, 2.10)	1.90 (1.40, 3.02)	<0.001
Lactate_mean, mmol/L	1.60 (1.10, 2.40)	1.60 (1.10, 2.20)	2.08 (1.44, 3.30)	<0.001
Lactate_max, mmol/L	1.60 (1.10, 2.40)	1.60 (1.00, 2.30)	1.83 (1.30, 3.30)	<0.001
Potassium_min, mmol/L	3.80 (3.50, 4.30)	3.80 (3.50, 4.20)	3.90 (3.40, 4.40)	0.089
Potassium_max, mmol/L	4.40 (4.00, 5.00)	4.40 (4.00, 4.90)	4.60 (4.10, 5.20)	<0.001
Potassium_mean, mmol/L	4.14 (3.80, 4.59)	4.12 (3.80, 4.53)	4.25 (3.80, 4.70)	0.005
Sodium_min, mmol/L	137.00 (134.00, 140.00)	137.00 (134.00, 140.00)	137.00 (133.00, 140.00)	0.021
Sodium_max, mmol/L	140.00 (137.00, 143.00)	140.00 (137.00, 143.00)	140.00 (136.00, 144.00)	0.714
Sodium_mean, mmol/L	139.00 (135.50, 141.50)	139.00 (135.83, 141.50)	138.45 (134.50, 142.00)	0.137
PT_min, sec	14.20 (12.50, 17.30)	13.90 (12.40, 16.40)	16.30 (13.40, 21.42)	<0.001
PT_max, sec	15.20 (13.10, 20.30)	14.80 (12.90, 19.00)	18.55 (14.50, 27.47)	<0.001
PT_mean, sec	14.75 (12.85, 18.90)	14.40 (12.70, 18.00)	17.58 (14.16, 24.51)	<0.001
APTT_min, sec	29.50 (26.10, 35.00)	28.80 (25.80, 33.50)	34.90 (29.10, 47.40)	<0.001
APTT_max, sec	33.30 (28.20, 46.30)	31.90 (27.70, 40.20)	46.20 (32.75, 68.30)	<0.001
APTT_mean, sec	31.80 (27.40, 40.60)	30.70 (26.95, 37.32)	40.95 (31.59, 57.27)	<0.001
INR_min	1.30 (1.10, 1.60)	1.20 (1.10, 1.50)	1.50 (1.20, 2.00)	<0.001
INR_max	1.40 (1.20, 1.90)	1.30 (1.20, 1.70)	1.70 (1.30, 2.60)	<0.001
INR_mean	1.33 (1.15, 1.73)	1.30 (1.15, 1.65)	1.60 (1.27, 2.28)	<0.001

The variceal bleeding study initially evaluated 92 candidate variables, with 70 demonstrating statistically significant associations (*p* < 0.001) upon rigorous screening. The feature variable selection results are presented in Table 4. To enhance the sensitivity of the ML model, we only incorporated 70 variables with *p* < 0.001.

#### 3.3.2 Machine learning model construction and evaluation

Hybrid approaches combining SMOTE-ENN and ADASYN techniques were employed to simultaneously address data imbalances. Subsequently, 12 ML algorithms were adopted to select the optimal ML models. The dual validation strategy incorporating both 5-fold cross validation (Table 6) and independent validation (Table 7) serves as robust safeguard against overfitting by providing multiple performance estimates and maintaining completely unseen data for final evaluation.

**Table 6:** 5-fold cross-validation of ML models prediction performance for ANGIB patients.

Models	Mean AUC	Mean accuracy	Mean precision	Mean recall	Mean F1 score
ADASYN					
Logistic regression	0.847 ± 0.016	0.782 ± 0.011	0.451 ± 0.018	0.734 ± 0.042	0.558 ± 0.025
Decision tree	0.722 ± 0.020	0.745 ± 0.009	0.396 ± 0.015	0.683 ± 0.073	0.500 ± 0.030
Random forest	0.842 ± 0.012	0.831 ± 0.009	0.616 ± 0.067	0.285 ± 0.019	0.388 ± 0.014
Gradient boosting	0.835 ± 0.014	0.847 ± 0.007	0.644 ± 0.032	0.423 ± 0.030	0.510 ± 0.025
AdaBoost	0.821 ± 0.016	0.837 ± 0.010	0.591 ± 0.034	0.426 ± 0.050	0.494 ± 0.041
XGBoost	0.826 ± 0.018	0.835 ± 0.017	0.587 ± 0.056	0.414 ± 0.063	0.484 ± 0.058
Naive Bayes	0.809 ± 0.018	0.792 ± 0.014	0.458 ± 0.028	0.558 ± 0.027	0.502 ± 0.020
SVM (RBF Kernel)	0.836 ± 0.017	0.799 ± 0.011	0.475 ± 0.023	0.649 ± 0.050	0.548 ± 0.028
LightGBM	0.831 ± 0.008	0.831 ± 0.007	0.551 ± 0.015	0.555 ± 0.053	0.552 ± 0.031
KNN	0.756 ± 0.015	0.830 ± 0.009	0.619 ± 0.063	0.241 ± 0.054	0.345 ± 0.062
Extra trees	0.848 ± 0.009	0.834 ± 0.009	0.566 ± 0.030	0.520 ± 0.030	0.541 ± 0.023
Voting classifier	0.853 ± 0.011	0.838 ± 0.012	0.580 ± 0.039	0.498 ± 0.050	0.535 ± 0.041
SMOTE-ENN					
Logistic regression	0.944 ± 0.018	0.870 ± 0.015	0.922 ± 0.022	0.865 ± 0.010	0.893 ± 0.012
Decision tree	0.906 ± 0.014	0.876 ± 0.004	0.919 ± 0.012	0.880 ± 0.008	0.899 ± 0.003
Random forest	0.985 ± 0.004	0.940 ± 0.012	0.934 ± 0.017	0.974 ± 0.007	0.953 ± 0.009
Gradient boosting	0.985 ± 0.002	0.948 ± 0.009	0.949 ± 0.009	0.968 ± 0.009	0.959 ± 0.007
AdaBoost	0.966 ± 0.006	0.911 ± 0.011	0.926 ± 0.017	0.932 ± 0.005	0.929 ± 0.008
XGBoost	0.983 ± 0.005	0.941 ± 0.014	0.945 ± 0.015	0.961 ± 0.009	0.953 ± 0.011
Naive Bayes	0.893 ± 0.025	0.808 ± 0.014	0.910 ± 0.023	0.770 ± 0.007	0.834 ± 0.010
SVM (RBF Kernel)	0.976 ± 0.008	0.924 ± 0.017	0.941 ± 0.017	0.938 ± 0.012	0.939 ± 0.013
LightGBM	0.986 ± 0.002	0.949 ± 0.007	0.958 ± 0.013	0.961 ± 0.008	0.959 ± 0.005
KNN	0.984 ± 0.006	0.938 ± 0.015	0.915 ± 0.018	0.995 ± 0.005	0.953 ± 0.011
Extra trees	0.978 ± 0.006	0.927 ± 0.014	0.948 ± 0.019	0.935 ± 0.006	0.941 ± 0.011
Voting classifier	0.984 ± 0.005	0.941 ± 0.015	0.946 ± 0.021	0.961 ± 0.007	0.953 ± 0.011

**Table 7:** Independent validation of ML models prediction performance for ANGIB Patients.

Models	Mean AUC	Mean accuracy	Mean precision	Mean recall	Mean F1 score
ADASYN					
Logistic regression	0.864 ± 0.012	0.781 ± 0.018	0.454 ± 0.025	0.787 ± 0.028	0.575 ± 0.024
Decision tree	0.732 ± 0.017	0.726 ± 0.024	0.376 ± 0.023	0.684 ± 0.049	0.484 ± 0.021
Random forest	0.861 ± 0.010	0.842 ± 0.007	0.635 ± 0.044	0.374 ± 0.046	0.468 ± 0.036
Gradient boosting	0.862 ± 0.014	0.847 ± 0.018	0.637 ± 0.084	0.448 ± 0.028	0.524 ± 0.039
AdaBoost	0.858 ± 0.017	0.841 ± 0.012	0.607 ± 0.062	0.464 ± 0.044	0.523 ± 0.028
XGBoost	0.859 ± 0.016	0.846 ± 0.011	0.641 ± 0.059	0.422 ± 0.053	0.506 ± 0.041
Naive Bayes	0.818 ± 0.019	0.790 ± 0.013	0.457 ± 0.023	0.631 ± 0.038	0.529 ± 0.025
SVM (RBF Kernel)	0.863 ± 0.011	0.817 ± 0.017	0.511 ± 0.033	0.727 ± 0.025	0.600 ± 0.029
LightGBM	0.869 ± 0.018	0.843 ± 0.017	0.577 ± 0.047	0.646 ± 0.031	0.608 ± 0.030
KNN	0.770 ± 0.021	0.838 ± 0.010	0.710 ± 0.092	0.237 ± 0.024	0.355 ± 0.033
Extra trees	0.871 ± 0.013	0.835 ± 0.012	0.559 ± 0.037	0.602 ± 0.036	0.578 ± 0.021
Voting classifier	0.877 ± 0.014	0.849 ± 0.014	0.608 ± 0.049	0.569 ± 0.040	0.586 ± 0.027
SMOTE-ENN					
Logistic regression	0.948 ± 0.007	0.872 ± 0.008	0.933 ± 0.009	0.856 ± 0.019	0.893 ± 0.008
Decision tree	0.936 ± 0.006	0.888 ± 0.004	0.940 ± 0.011	0.876 ± 0.015	0.907 ± 0.004
Random forest	0.988 ± 0.005	0.954 ± 0.007	0.950 ± 0.010	0.978 ± 0.014	0.964 ± 0.006
Gradient boosting	0.990 ± 0.003	0.958 ± 0.005	0.956 ± 0.006	0.978 ± 0.011	0.967 ± 0.004
AdaBoost	0.972 ± 0.007	0.925 ± 0.007	0.940 ± 0.010	0.941 ± 0.013	0.940 ± 0.006
XGBoost	0.988 ± 0.004	0.955 ± 0.009	0.955 ± 0.008	0.974 ± 0.013	0.964 ± 0.008
Naive Bayes	0.902 ± 0.013	0.811 ± 0.009	0.919 ± 0.011	0.765 ± 0.012	0.835 ± 0.008
SVM (RBF Kernel)	0.984 ± 0.006	0.945 ± 0.007	0.959 ± 0.012	0.954 ± 0.006	0.956 ± 0.005
LightGBM	0.991 ± 0.004	0.963 ± 0.006	0.965 ± 0.008	0.976 ± 0.012	0.970 ± 0.005
KNN	0.993 ± 0.004	0.962 ± 0.006	0.946 ± 0.007	0.997 ± 0.003	0.971 ± 0.004
Extra trees	0.983 ± 0.004	0.940 ± 0.006	0.958 ± 0.006	0.946 ± 0.007	0.952 ± 0.005
Voting classifier	0.987 ± 0.005	0.958 ± 0.005	0.958 ± 0.009	0.975 ± 0.009	0.966 ± 0.004

Through comprehensive evaluation of key parameters (including accuracy, precision, recall, and F1-score), the Gradient Boosting model exhibited excellent prediction performance with AUC of 0.985 ± 0.002, accuracy of 0.948 ± 0.009, precision of 0.949 ± 0.009, recall of 0.968 ± 0.009, and F1-score of 0.959 ± 0.007, respectively. Thus, GB model demonstrated superior performance and was consequently selected as the excellent predictive framework.

#### 3.3.3 Machine learning model performance

As represented in Table 6, the SMOTE-ENN imbalance-handling technique achieves superior performance across nearly all metrics. Consequently, SMOTE-ENN was finally selected as the optimal algorithm, outperforming the ADASYN technique. The consistency performance observed in both 5-CV and IV confirmed that SMOTE-ENN effectively mitigates class imbalance issue without generalizing overfitting, making it the most reliable resampling approach.

**Figure 4:**
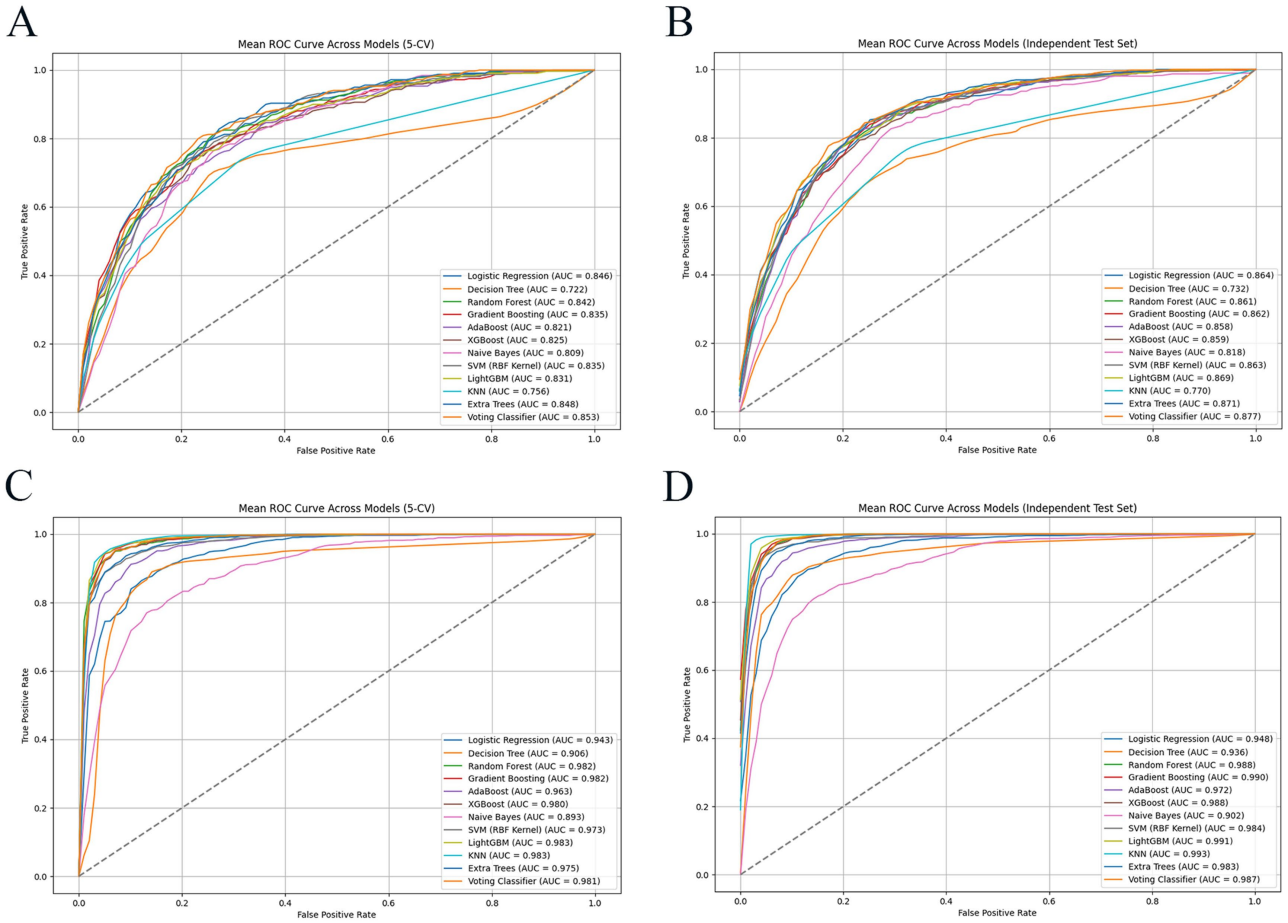
AUC of different machine learning models for ANGIB patients in 5 CV and IV. **(A)** The ROC of models by ADASYN technique in 5 CV; **(B)** the ROC of models by ADASYN technique in IV; **(C)** the ROC of models by SMOTE-ENN technique in 5 CV; **(D)** the ROC of models by SMOTE-ENN technique in IV. SMOTE-ENN: Synthetic Minority Over-sampling Technique-Edited Nearest Neighbors; ADASYN: Adaptive Synthetic Sampling.

Regarding the ML model performance, the AUC values varied significantly across different algorithms when using the ADASYN and SMOTE-ENN techniques, as evaluated through both 5-CV and IV in Figure 4. The ET model emerged as the optimal choice due to the highest AUC values and overall excellent performance, as detailed in Section 3.2.2.

#### 3.3.4 Machine learning model SHAP explainable

To directly display the weights of each feature variable and its predictive values of the GB model, SHAP (Shapley Additive exPlanations) algorithms were performed to visualize variables. The SHAP summary plot contained average SHAP values of variables and a representation of each feature’s contribution with SHAP values. The top 20 feature variables were GCS score, vasoactive drugs, acute kidney failure, AIMS65 score, APACHE-II score, mechanical ventilation, lactate_min, chronic liver disease, APTT_min, APTT_max, potassium_max, acute heart failure, anticoagulants, albumin_max, APTT_mean, BUN_miin, sepsis, respiratory rate_max, WBC_min, and heart rate_max.

**Figure 5:**
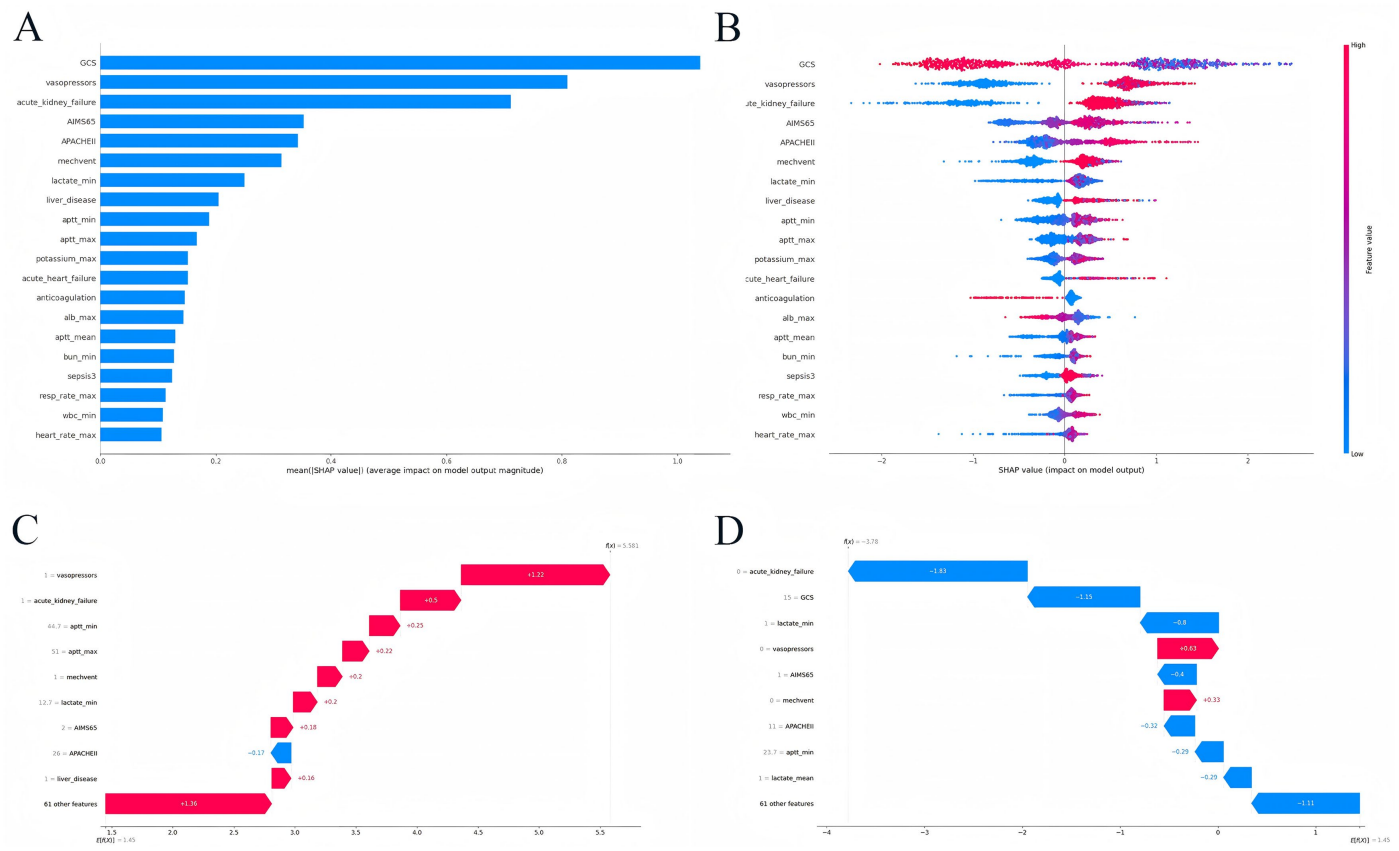
SHAP explainable of the gradient boosting prediction model. **(A)** The SHAP feature importance plot; **(B)** the SHAP summary plot; **(C)** the ML explainability of positive patient; **(D)** the ML explainability of negative patient.

The SHAP feature importance plot indicated top 20 feature variable weight of the optimal GB model in mortality prediction (Figure 5A). In the SHAP summary plot, each point represents the SHAP value of corresponding feature variable for given sample (Figure 5B). Similarly, points trending toward red color indicate higher feature values, while those approaching blue color denote lower feature values.

#### 3.3.5 Machine learning Explainability for patients

The SHAP explains model predictions by quantifying feature contributions, visualized via waterfall plots. The positive patient was randomly selected in Figure 5C. The base value of the GB model is E *f*(*x*) = 1.36, the vasoactive drugs corresponding to *f*(*x*) = 1.22 and previous acute kidney failure corresponding to *f*(*x*) = 0.50. Similarly, other feature variables correspond to f(x) values. As described in Figure 5C, the final *f*(*x*) was 5.581; therefore, the patient was positive case representative.

Similarly, the negative patient was randomly selected in Figure 5D. The base value of the GB model is E *f*(*x*) = −1.11, and the patient had not acute kidney failure, corresponding to *f*(*x*) = −1.83; lactate min value was 1.0 mmol/L corresponding to *f*(*x*) = −0.80. Similarly, other feature variables correspond to *f*(*x*) values. As described in Figure 5D, the final *f*(*x*) was-3.78; therefore, the patient was negative case representative.

## 4 Discussion

Despite significant advancements in the prevention and treatment of AUGIB, the prognosis still remains great challenge, especially during ICU hospitalization (33). AUGIB patients constantly presented with massive bleeding, persistent hematemesis, melena, and even with active bleeding. Due to underlying circulatory failure and the possibility of MODS, high-risk patients often require ICU intensive bundle therapy (2). Multidisciplinary collaboration, fluid resuscitation, blood transfusion, correction of coagulopathy, and early endoscopic interventions all contribute to the outcomes (34–36). However, not all patients admitted to the ICU received emergency endoscopy, pharmacological hemostasis, blood transfusion, interventional therapy, or surgical intervention, it was judged by conditions. Previous scores, such as AIMS65, Rockall, and GBS, could be utilized as risk stratification tools. However, the score’s accuracy, sensitivity, and specificity were unsatisfactory, ranging from 70 to 80% (37). The APACHE-II score may be used for prediction in ICU. However, relevant studies are relatively limited (38).

The AUGIB patient mortality is associated with multiple factors, including patient age, medical history, personal history, laboratory tests, and vital signs. Therefore, an integrated model with various variables is desperately needed. Variceal and non-variceal, as major categories of acute upper gastrointestinal hemorrhage, substantially influence clinical outcomes (39). Machine learning models are emerging as powerful tools by AI algorithms, which can achieve high accuracy and automated decision-making with highly adaptable and predictive power (40). Zhao X model was especially for non-variceal GIB patients (12), while the Agarwal S model was used for esophageal varices patients (41). The Kou Y prediction model was designed for GIB patients with acute myocardial infarction (AMI) (42). Thus, we aimed to construct ML model for AUGIB patients based on varice and non-varice subtypes. The MIMC-IV database was real-world clinical data from the ICU of Beth Israel Deaconess Medical Center (BIDMC), open access with millions of electronic health records including structured data and non-structured data, physiological waveform data, and time series data, which was the ideal resource for the ML model (43). The dynamic initial value, the minimum, max, and mean value give more accurate data for prediction. Thus, multiple values of vital signs and laboratory results were integrated into the ML model.

The ET model exhibited excellent performance for AVIGB patients with AUC of 0.996 ± 0.007, accuracy of 0.966 ± 0.009, precision of 0.957 ± 0.024, recall of 0.988 ± 0.012, and F1-score of 0.972 ± 0.007; the GB model was the optimal for ANGIB patients with AUC of 0.985 ± 0.002, accuracy of 0.948 ± 0.009, precision of 0.949 ± 0.009, recall of 0.968 ± 0.009, and F1-score of 0.959 ± 0.007, respectively. In other words, both prediction models can accurately identify positive patients while correctly recognizing negative patients better than previous ML models. Further analysis of the risk factors ranking in the top 10 revealed that vasoactive drugs, GCS score, AIMS65 score, anticoagulation, and SpO_2_ were the most related variables.

(1) Vasoactive agents: If patients present with persistent bleeding or hemodynamic instability or with comorbidities such as cirrhosis, renal insufficiency, or heart failure, vasoactive drugs should be considered to maintain blood pressure and improve tissue perfusion. AGA Clinical Practice in 2024 suggested that vasoactive drugs should be initiated as soon as the diagnosis of variceal hemorrhage should be continued for 2 to 5 days to prevent early rebleeding (44). As described in previous research, vasoactive agents might be closely related to mortality (45). The use of vasoactive agents indicates hypotension, shock, or circulatory failure, all of which are associated with poor outcomes in AUGIB patients. (2) GCS score: The GCS (Glasgow Coma Scale) score consists of three components: verbal response, eye-opening response, and motor response, which collectively reflect the level of consciousness. Patients with hemodynamic instability, advanced cirrhosis with hepatic encephalopathy (HE), or severe cardiac or renal dysfunction may present with varying degrees of consciousness, leading to diverse GCS scores. Qiu W’s research indicated that a higher GCS score was associated with an increased risk of GIB patients (45). AUGIB patients in ICU have a higher proportion of advanced cirrhosis, chronic renal insufficiency, or congestive heart failure; the lower GCS scores were independent predictive factors of mortality (46). (3) AIMS 65 score: The AIMS65 developed in 2011 by Saltzman JR was simple and easy to implement without endoscopy result (47). It focused on the evaluation of in-hospital mortality with high accuracy (6). The AIMS65 scale also had good predictability and was suitable for rapid preliminary evaluation at the outset (48). The prediction value of AIMS65 was also confirmed by the ML model, as expected. (4) Anticoagulants: The AGC 2020 guideline stresses the association between anticoagulants and antiplatelets in acute GIB patients, and the administration of fresh frozen plasma (FFP) can significantly impact prognosis (49). Gastrointestinal bleeding patients who have coagulopathies or are on oral anticoagulants or antiplatelet agents often face a high risk of massive bleeding or rebleeding due to deficiencies in coagulation factors and prolonged hemostasis. (5) SpO_2_: SpO_2_ is a standard indicator for evaluating oxygenation and is characteristic of non-invasive and continuous measures. The minimum SpO_2_ value indicates the onset of hypoxemia, which reflects inadequate tissue and organ perfusion levels and a decline in cardiorespiratory function (10). In addition, reduced SpO_2_ is a risk prognostic factor for disease progression (50). The minimum of SpO_2_ was an independent risk factor in our study, consistent with other researchers (10, 51).

While our ML model demonstrated excellent prediction performance with large-scale data, several limitations should be acknowledged. First, as a retrospective study, it was inherently subject to selection biases or system biases, which may have led to the omission of specific crucial parameters. Second, the MIMIC-IV database is regarded as a signal center medical unit; external validation is required further to confirm the prediction value of the optimal ML models. Third, the etiology of GIB patients is diverse. In this study, no diagnosis-related subgroup analysis was conducted, which may have affected the accuracy to some extent.

## 5 Conclusion

In this study, we developed prediction models especially for AVGIB and ANGIB hospital ICU patients based on as many as 12 standard algorithms. Considering the imbalanced dataset of real-world patients, the SMOTE-ENN technique was performed to improve model performance and optimize evaluation metrics. When compared with key parameters, gradient boosting and extremely randomized tree both ranked first with excellent performance by integrating feature variables. The SHAP plot visualization displays feature variables by weight: vasoactive drugs, GCS score, AIMS65 score, anticoagulants, and SpO_2_. Most importantly, two website prognostic prediction platforms were developed to enhance clinical accessibility: the ET model for AVGIB patients available at https://10zr656do5281.vicp.fun while the GB model for ANGIB patients accessible at http://10zr656do5281.vicp.fun.

The models provide valuable decision support for clinicians, enabling the early identification of at-risk patients, timely initiation of endoscopy, correction of coagulation dysfunction, fluid resuscitation, and combined with interventional or even surgery to reduce mortality and improve outcomes.

## Data Availability

The original contributions presented in the study are included in the article/Supplementary material, further inquiries can be directed to the corresponding author.
