# Supplementary material for: The future of critical care: AI-powered mortality prediction for acute variceal gastrointestinal bleeding and acute non-variceal gastrointestinal bleeding patients
Source: Front Med (Lausanne). 2025 May 16;12:1580094. doi: 10.3389/fmed.2025.1580094 (PMC12122533; doi:10.3389/fmed.2025.1580094)
Supplement: Supplementary file 1 [file Table_1.doc]

| **Supplement Table 1** The survival and non-survival AUGIB patients clinical characteristic in MIMIC-IV database | | | | |
| --- | --- | --- | --- | --- |
| **Variables** | **Overall (n=3050)** | **Survival (n=2466)** | **Non-survival (n=584)** | ***P* value** |
| **Demographics** |  |  |  |  |
| Age, y | 64.81 (54.07, 77.31) | 64.61 (53.84, 77.37) | 65.35 (54.53, 77.04) | 0.573 |
| Male, n (%) | 1883 (61.74) | 1501 (60.87) | 382 (65.41) | 0.042 |
| **Medical history** |  |  |  |  |
| Anticoagulants, n (%) | 507 (16.62) | 442 (17.92) | 65 (11.13) | ＜0.001 |
| Antiplatelet agents, n (%) | 1002 (32.85) | 830 (33.66) | 172 (29.45) | 0.052 |
| Proton Pump Inhibitors, n (%) | 2941 (96.43) | 2377 (96.39) | 564 (96.58) | 0.829 |
| **Intervention measures** |  |  |  |  |
| Vasoactive drugs, n (%) | 1030 (33.77) | 637 (25.83） | 393 (67.29) | ＜0.001 |
| CRRT, n (%) | 209 (6.85) | 98 (3.97) | 111 (19.01) | ＜0.001 |
| Mechanical Ventilation, n (%) | 1319 (43.25) | 917 (37.19) | 402 (68.84) | ＜0.001 |
| **Vital Sign** |  |  |  |  |
| Heart rate_min, bmp | 72.00 (63.00, 84.00) | 72.00 (63.00, 83.00) | 76.00 (62.75, 89.00) | ＜0.001 |
| Heart rate_max, bmp | 105.00 (91.00, 119.00) | 104.00 (90.00, 118.00) | 110.00 (97.00, 125.00) | ＜0.001 |
| Heart rate_mean, bmp | 86.88 (75.93, 98.21) | 85.65 (75.26, 97.09) | 91.41 (80.15, 103.68) | ＜0.001 |
| Respiratory rate_min, bmp | 12.00 (10.00, 15.00) | 12.00 (10.00, 14.00) | 13.00 (10.00, 15.12) | ＜0.001 |
| Respiratory rate_max, bmp | 27.00 (24.00, 31.00) | 27.00 (23.00, 31.00) | 29.00 (25.00, 33.00) | ＜0.001 |
| Respiratory rate_mean, bmp | 18.59 (16.43, 21.33) | 18.32 (16.28, 20.82) | 19.92 (17.23, 23.57) | ＜0.001 |
| SPB_min, mmHg | 90.00 (80.00, 100.00) | 91.00 (82.00, 102.00) | 83.00 (75.00, 92.00) | ＜0.001 |
| SPB_max, mmHg | 143.00 (128.00, 160.00) | 145.00 (129.00, 161.00) | 138.00 (122.00, 153.00) | ＜0.001 |
| SPB_mean, mmHg | 113.74 (104.07, 126.71) | 115.80 (105.96, 128.42) | 106.81 (99.00, 116.50) | ＜0.001 |
| DBP_min, mmHg | 45.00 (38.00, 53.00) | 46.00 (38.00, 53.00) | 42.00 (34.00, 49.00) | ＜0.001 |
| DBP_max, mmHg | 86.00 (75.00, 99.00) | 87.00 (76.00, 100.00) | 82.00 (70.00, 96.25) | ＜0.001 |
| DBP_mean, mmHg | 61.48 (54.70, 69.67) | 62.55 (55.73, 70.48) | 57.73 (51.52, 65.17) | ＜0.001 |
| MBP_min, mmHg | 58.00 (50.00, 65.00) | 59.00 (51.00, 66.00) | 53.00 (46.00, 60.25) | ＜0.001 |
| MBP_max, mmHg | 100.00 (88.00, 113.00) | 100.00 (89.00, 114.00) | 96.00 (86.00, 111.25) | ＜0.001 |
| MBP_mean, mmHg | 75.28 (68.80, 83.39) | 76.38 (69.70, 84.39) | 71.16 (65.75, 78.07) | ＜0.001 |
| SpO2_min, % | 93.00 (90.00, 95.00) | 93.00 (91.00, 95.00) | 92.00 (89.00, 94.25) | ＜0.001 |
| SpO2_max, % | 100.00 (100.00, 100.00) | 100.00 (100.00, 100.00) | 100.00 (100.00, 100.00) | 0.866 |
| SpO2_mean, % | 97.42 (96.08, 98.65) | 97.50 (96.25, 98.66) | 97.02 (95.41, 98.61) | ＜0.001 |
| **Previous history** |  |  |  |  |
| Myocardial Infarction, n (%) | 472 (15.48) | 359 (14.56) | 113 (19.35) | 0.004 |
| Congestive Heart Failure, n (%) | 911 (29.87) | 702 (28.47) | 209 (35.79) | 0.001 |
| Hypertension, n (%) | 803 (26.33) | 684 (27.74) | 119 (20.38) | ＜0.001 |
| Diabetes, n (%) | 973 (31.90) | 807 (32.73) | 166 (28.42) | 0.045 |
| Atrial Fibrillation, n (%) | 788 (25.84) | 615 (24.94) | 173 (29.62) | 0.020 |
| Chronic Kidney Disease, n (%) | 851 (27.90) | 672 (27.25) | 179 (30.65) | 0.100 |
| COPD, n (%) | 321 (10.52) | 243 (9.85) | 78 (13.36) | 0.013 |
| Chronic Liver Disease, n (%) | 1350 (44.26) | 1015 (41.16) | 335 (57.36) | ＜0.001 |
| **Blood Transfusion** |  |  |  |  |
| Red Blood Cells | 1912 (62.69) | 1517 (61.52) | 395 (67.64) | 0.006 |
| Plasma | 752 (24.66) | 491 (19.91) | 261 (44.69) | ＜0.001 |
| Platelets | 548 (17.97) | 351 (14.23) | 197 (33.73) | ＜0.001 |
| Albumin | 612 (20.1) | 387 (15.69) | 225(38.52) | ＜0.001 |
| **Related Scores** |  |  |  |  |
| APACHE-II | 12.00 (9.00, 16.00) | 11.00 (9.00, 15.00) | 16.00 (12.00, 21.00) | ＜0.001 |
| GBS | 13.00 (10.00, 15.00) | 13.00 (10.00, 15.00) | 13.00 (10.00, 15.00) | ＜0.001 |
| AIMS65 | 2.00 (1.00, 3.00) | 2.00 (1.00, 3.00) | 3.00 (2.00, 3.00) | ＜0.001 |
| Rockall score | 7.00 (6.00, 7.00) | 7.00 (5.00, 7.00) | 7.00 (6.00, 8.00) | ＜0.001 |
| Shock index | 0.76 (0.63, 0.92) | 0.75 (0.62, 0.90) | 0.85 (0.70, 1.00) | ＜0.001 |
| GCS score | 14.00 (11.00, 15.00) | 14.00 (13.00, 15.00) | 11.00 (6.00, 14.00) | ＜0.001 |
| **Laboratory Result** |  |  |  |  |
| WBC_min, 10^9^/L | 8.20 (5.50, 12.00) | 7.95 (5.40, 11.40) | 10.20 (6.17, 14.72) | ＜0.001 |
| WBC_max, 10^9^/L | 11.70 (7.80, 17.10) | 11.20 (7.53, 16.10) | 14.30 (9.70, 20.82) | ＜0.001 |
| WBC_mean, 10^9^/L | 10.02 (6.73, 14.35) | 9.60 (6.57, 13.54) | 12.20 (7.99, 17.73) | ＜0.001 |
| RBC_min, 10^12^/L | 2.74 (2.35, 3.22) | 2.76 (2.38, 3.25) | 2.65 (2.24, 3.09) | ＜0.001 |
| RBC_max, 10^12^/L | 3.28 (2.90, 3.77) | 3.30 (2.92, 3.78) | 3.19 (2.82, 3.71) | 0.009 |
| RBC_mean, 10^12^/L | 3.00 (2.67, 3.47) | 3.03 (2.69, 3.48) | 2.90 (2.56, 3.38) | 0.001 |
| Hb_min, mg/dL | 8.20 (7.00, 9.70) | 8.20 (7.10, 9.70) | 8.10 (7.00, 9.60) | 0.259 |
| Hb_max, mg/dL | 9.90 (8.70, 11.30) | 9.90 (8.70, 11.30) | 10.00 (8.60, 11.30) | 0.656 |
| Hb_mean, mg/dL | 9.03 (7.97, 10.37) | 9.05 (7.97, 10.37) | 8.95 (7.97, 10.35) | 0.751 |
| PLT_min, 10^9^/L | 134.00 (76.00, 210.00) | 141.00 (83.00, 215.00) | 106.00 (55.00, 183.00) | ＜0.001 |
| PLT_max, 10^9^/L | 180.00 (111.00, 265.00) | 188.00 (118.00, 271.00) | 149.00 (88.75, 229.25) | ＜0.001 |
| PLT_mean, 10^9^/L | 155.63 (94.07, 231.67) | 163.35 (100.68, 237.00) | 123.55 (71.65, 206.31) | ＜0.001 |
| ALT_min, U/L | 23.00 (14.00, 42.00) | 22.00 (14.00, 39.00) | 30.00 (17.00, 63.00) | ＜0.001 |
| ALT_max, U/L | 25.00 (15.00, 50.00) | 24.00 (15.00, 44.00) | 34.00 (19.75, 82.25) | ＜0.001 |
| ALT_mean, U/L | 24.00 (15.00, 46.94) | 23.00 (14.00, 42.00) | 32.00 (18.00, 75.00) | ＜0.001 |
| AST_min, U/L | 35.00 (21.00, 77.00) | 32.00 (20.00, 66.00) | 53.00 (26.00, 123.25) | ＜0.001 |
| AST_max, U/L | 40.00 (22.00, 91.00) | 36.00 (21.00, 80.00) | 61.50 (30.00, 181.25) | ＜0.001 |
| AST_mean, U/L | 38.00 (21.75, 84.95) | 35.00 (21.00, 74.33) | 57.00 (29.00, 165.25) | ＜0.001 |
| Albumin_min, mg/dL | 3.00 (2.60, 3.43) | 3.06 (2.66, 3.50) | 2.80 (2.30, 3.29) | ＜0.001 |
| Albumin_max, mg/dL | 3.10 (2.67, 3.50) | 3.10 (2.70, 3.56) | 2.90 (2.40, 3.32) | ＜0.001 |
| Albumin_mean, mg/dL | 3.05 (2.60, 3.50) | 3.10 (2.70, 3.50) | 2.85 (2.40, 3.30) | ＜0.001 |
| TBIL_min, mg/dL | 0.92 (0.50, 2.44) | 0.84 (0.50, 1.96) | 2.00 (0.62, 7.60) | ＜0.001 |
| TBIL_max, mg/dL | 1.10 (0.53, 3.00) | 1.00 (0.50, 2.40) | 2.50 (0.70, 9.03) | ＜0.001 |
| TBIL_mean, mg/dL | 1.05 (0.50, 2.79) | 0.95 (0.50, 2.20) | 2.20 (0.70, 8.31) | ＜0.001 |
| BUN_min, mg/dL | 25.00 (15.00, 42.00) | 23.00 (14.00, 39.00) | 33.00 (20.00, 54.50) | ＜0.001 |
| BUN_max, mg/dL | 30.00 (18.00, 52.00) | 29.00 (18.00, 48.00) | 40.00 (24.50, 65.00) | ＜0.001 |
| BUN_mean, mg/dL | 27.50 (17.00, 47.33) | 26.00 (16.00, 43.50) | 37.00 (22.83, 60.38) | ＜0.001 |
| Creatinine_min, mg/dL | 1.00 (0.70, 1.70) | 1.00 (0.70, 1.60) | 1.40 (0.80, 2.30) | ＜0.001 |
| Creatinine_max, mg/dL | 1.20 (0.80, 2.10) | 1.10 (0.80, 1.90) | 1.80 (1.05, 3.00) | ＜0.001 |
| Creatinine_mean, mg/dL | 1.12 (0.77, 1.90) | 1.05 (0.75, 1.73) | 1.62 (1.00, 2.55) | ＜0.001 |
| Lactate_min, mmol/L | 1.60 (1.10, 2.30) | 1.60 (1.10, 2.20) | 2.00 (1.40, 3.30) | ＜0.001 |
| Lactate_max, mmol/L | 1.60 (1.20, 2.45) | 1.60 (1.10, 2.25) | 2.15 (1.45, 3.40) | ＜0.001 |
| Lactate_mean, mmol/L | 1.60 (1.10, 2.50) | 1.60 (1.00, 2.30) | 1.90 (1.30, 3.42) | ＜0.001 |
| Potassium_min, mmol/L | 3.90 (3.50, 4.30) | 3.90 (3.50, 4.30) | 3.90 (3.40, 4.40) | 0.106 |
| Potassium_max, mmol/L | 4.40 (4.00, 5.00) | 4.40 (4.00, 5.00) | 4.60 (4.10, 5.30) | ＜0.001 |
| Potassium_mean, mmol/L | 4.15 (3.80, 4.60) | 4.14 (3.80, 4.55) | 4.25 (3.80, 4.73) | 0.002 |
| Sodium_min,mmol/L | 137.00 (134.00, 140.00) | 137.00 (134.00, 140.00) | 136.00 (132.00, 140.00) | 0.003 |
| Sodium_max,mmol/L | 140.00 (137.00, 143.00) | 140.00 (137.00, 143.00) | 140.00 (135.00, 144.00) | 0.406 |
| Sodium_mean,mmol/L | 138.78 (135.50, 141.50) | 139.00 (135.75, 141.50) | 138.29 (134.00, 142.00) | 0.041 |
| PT_min, sec | 14.60 (12.80, 18.10) | 14.30 (12.60, 17.10) | 17.40 (13.90, 21.80) | ＜0.001 |
| PT_max, sec | 16.00 (13.40, 21.20) | 15.40 (13.20, 19.70) | 20.15 (15.20, 28.50) | ＜0.001 |
| PT_mean, sec | 15.40 (13.17, 19.69) | 14.90 (12.95, 18.45) | 18.86 (14.65, 25.61) | ＜0.001 |
| APTT_min, sec | 30.20 (26.40, 36.00) | 29.30 (26.10, 34.20) | 36.00 (29.88, 46.98) | ＜0.001 |
| APTT_max, sec | 33.90 (28.60, 46.20) | 32.60 (28.10, 40.60) | 47.35 (33.95, 69.78) | ＜0.001 |
| APTT_mean, sec | 32.42 (27.84, 41.06) | 31.30 (27.35, 37.76) | 42.95 (32.56, 57.41) | ＜0.001 |
| INR_min | 1.30 (1.20, 1.70) | 1.30 (1.10, 1.60) | 1.60 (1.30, 2.00) | ＜0.001 |
| INR_max | 1.45 (1.20, 2.00) | 1.40 (1.20, 1.80) | 1.90 (1.40, 2.70) | ＜0.001 |
| INR_mean | 1.40 (1.20, 1.80) | 1.35 (1.18, 1.70) | 1.74 (1.30, 2.39) | ＜0.001 |
